# Exponential Time Decay of Solutions to Reaction-Cross-Diffusion Systems of Maxwell–Stefan Type

**DOI:** 10.1007/s00205-019-01439-9

**Published:** 2019-08-01

**Authors:** Esther S. Daus, Ansgar Jüngel, Bao Quoc Tang

**Affiliations:** 1 Institute for Analysis and Scientific Computing, Vienna University of Technology, Wiedner Hauptstraße 8–10, 1040 Wien, Austria; 2 Institute of Mathematics and Scientific Computing, University of Graz, Heinrichstrasse 36, 8010 Graz, Austria

## Abstract

The large-time asymptotics of weak solutions to Maxwell–Stefan diffusion systems for chemically reacting fluids with different molar masses and reversible reactions are investigated. The diffusion matrix of the system is generally neither symmetric nor positive definite, but the equations admit a formal gradient-flow structure which provides entropy (free energy) estimates. The main result is the exponential decay to the unique equilibrium with a rate that is constructive up to a finite-dimensional inequality. The key elements of the proof are the existence of a unique detailed-balance equilibrium and the derivation of an inequality relating the entropy and the entropy production. The main difficulty comes from the fact that the reactions are represented by molar fractions while the conservation laws hold for the concentrations. The idea is to enlarge the space of *n* partial concentrations by adding the total concentration, viewed as an independent variable, thus working with $n+1$ variables. Further results concern the existence of global bounded weak solutions to the parabolic system and an extension of the results to complex-balance systems.

## Introduction

The analysis of the large-time behavior of dynamical networks is important to the understanding of their stability properties. Of particular interest are reversible chemical reactions interacting with diffusion. While there is a vast literature on the large-time asymptotics of reaction–diffusion systems, much less is available for reaction systems with cross-diffusion terms. Such systems arise naturally in multicomponent fluid modeling and population dynamics [38]. In this paper, we prove the exponential decay of solutions to reaction-cross-diffusion systems of Maxwell–Stefan form by combining recent techniques for cross-diffusion systems [37] and reaction–diffusion equations [25]. The main feature of our result is that the decay rate is constructive up to a finite-dimensional inequality and that the result holds for detailed-balance or complex-balance systems.

### Model Equations

We consider a fluid consisting of *n* constituents $A_i$ with mass densities $\rho _i(z,t)$ and molar masses $M_i$, which are diffusing according to the diffusive fluxes ${\varvec{j}}_i(z,t)$ and reacting in the following reversible reactions:

$$\begin{aligned} \alpha _1^a A_1 + \cdots + \alpha _n^a A_n \leftrightharpoons \beta _1^a A_1 + \cdots + \beta _n^a A_n \quad \text{ for } a=1,\ldots ,N, \end{aligned}$$

where $\alpha _i^a$ and $\beta _i^a$ are the stoichiometric coefficients. The evolution of the fluid is assumed to be governed by partial mass balances with Maxwell–Stefan relations for the diffusive fluxes

1 $$\begin{aligned} \partial _t\rho _i + {\text {div}}{\varvec{j}}_i = r_i({\varvec{x}}), \quad \nabla x_i = -\sum _{j=1}^n\frac{\rho _j{\varvec{j}}_i-\rho _i{\varvec{j}}_j}{c^2M_iM_jD_{ij}}, \quad i=1,\ldots ,n, \end{aligned}$$

where $x_i=c_i/c$ are the molar fractions, $c_i=\rho _i/M_i$ the partial concentrations, $M_i$ the molar masses, $c=\sum _{i=1}^n c_i$ the total concentration, and $D_{ij}=D_{ji}>0$ are the diffusivities. The physical quantities are summarized in Table 1. The reactions are described by the mass production terms $r_i$ depending on ${\varvec{x}}=(x_1,\ldots ,x_n)$ using mass-action kinetics:

2 $$\begin{aligned} r_i({\varvec{x}}) = M_i\sum _{a=1}^N(\beta _i^a-\alpha _i^a) (k_f^a{\varvec{x}}^{{\varvec{\alpha ^a}}} - k_b^a{\varvec{x}}^{{\varvec{\beta ^a}}}) \quad \text{ with } {\varvec{x}}^{{\varvec{\alpha ^a}}}:=\prod _{i=1}^n x_i^{\alpha _i^a}, \end{aligned}$$

where $k_f^a>0$ and $k_b^a>0$ are the forward and backward reaction rate constants, respectively, and ${\varvec{\alpha }}^a=(\alpha _1^a,\ldots ,\alpha _n^a)$ and ${\varvec{\beta }}^a=(\beta _1^a,\ldots ,\beta _n^a)$ with $\alpha _i^a$, $\beta _i^a\in \{0\} \cup [1,\infty )$ are the vectors of the stoichiometric coefficients.

**Table 1:** Overview of the physical quantities

$\rho _i$	: partial mass density of the *i*th species
$\rho =\sum _{i=1}^n \rho _i$	: total mass density
${\varvec{j}}_i$	: partial particle flux of the *i*th species
$M_i$	: molar mass of the *i*th species
$c_i = \rho _i/M_i$	: partial concentration of the *i*th species
$c=\sum _{i=1}^n c_i$	: total concentration
$x_i = c_i/c$	: molar fraction

Equations (1) are solved in the bounded domain $\Omega \subset {\mathbb {R}}^d$ ($d\geqslant 1$) subject to the no-flux boundary and initial conditions

3 $$\begin{aligned} {\varvec{j}}_i\cdot \nu =0 \text{ on } \partial \Omega ,\quad \rho _i(\cdot ,0)=\rho _i^0 \quad \text{ in } \Omega ,\ i=1,\ldots ,n. \end{aligned}$$

To simplify, we assume that $\Omega$ has unit measure, i.e. $|\Omega | = 1$.

System (1)–(2) models a multicomponent fluid in an isothermal regime with vanishing barycentric velocity. The Maxwell–Stefan diffusion system models diffusive transport of multicomponent diffusion and was first introduced by Maxwell [42] and Stefan [52]. Since then, the range of applications goes from respiratory airways [8] to dialysis, electrolysis, sedimentation, ion exchange or ultrafiltration [54, 57]. Equation (1) for $\nabla x_i$ can be derived from the Boltzmann equations for mixtures in the diffusive limit and with well-prepared initial conditions [9, 35, 36] or from the reduced force balances with the partial momentum productions being proportional to the partial velocity differences [6, Section 14]. It can also be derived from a kinetic model of a reacting sphere system [3] or by careful exploitation of the entropy principle [6, Sections 7–8]. Concerning the isothermal regime, we remark that, even though the chemical reactions usually modify the temperature of the system, there exist situations in which a heat bath is sufficiently efficient for keeping the whole system at the same temperature. For more details, we refer the interested reader to, e.g., the invention [56], which designs an engine for compressing gaseous fluids isothermally. Moreover, our analysis of the isothermal case can be used as a starting point in investigating more complex, non-isothermal systems.

We assume that the total mass is conserved and that the mixture is at rest, i.e., $\sum _{i=1}^n\rho _i=1$ and $\sum _{i=1}^n{\varvec{j}}_i=0$. This implies that

4 $$\begin{aligned} \sum _{i=1}^n r_i({\varvec{x}})=0 \quad \text{ for } \text{ all } {\varvec{x}}=(x_1,\ldots ,x_n)\in {\mathbb {R}}_+^n, \end{aligned}$$

where ${\mathbb {R}}_+=(0,\infty )$. Furthermore, we assume that the system of reactions satisfies a detailed-balance condition, meaning that there exists a positive homogeneous equilibrium ${\varvec{x}}_\infty \in {\mathbb {R}}_+^n$ such that

5 $$\begin{aligned} k_f^a{\varvec{x}}_\infty ^{{\varvec{\alpha }}^a} = k_b^a{\varvec{x}}_\infty ^{{\varvec{\beta }}^a} \quad \text{ for } \text{ all } a=1,\ldots ,N. \end{aligned}$$

Roughly speaking, a system is under detailed balance if any forward reaction is balanced by the corresponding backward reaction at equilibrium. Condition (5) does not give a unique but instead a manifold of detailed-balance equilibria,

6 $$\begin{aligned} \mathcal {E}= \big \{{\varvec{x}}_\infty \in {\mathbb {R}}_+^n:\ k_f^a{\varvec{x}}_\infty ^{{\varvec{\alpha }}^a} = k_b^a{\varvec{x}}_\infty ^{{\varvec{\beta }}^a}\quad \text{ for } \text{ all } a=1,\ldots ,N\big \}. \end{aligned}$$

To uniquely identify the detailed-balance equilibrium, we need to take into account the conservation laws (meaning that certain linear combinations of the concentrations are constant in time). This is discussed in detail below. We are also able to consider complex-balance systems; see Section 5.

The aim of this paper is to prove that under these conditions, there exists a unique positive detailed-balance (or complex-balance) equilibrium ${\varvec{x}}_\infty =(x_{1\infty },\ldots ,x_{n\infty })\in {\mathbb {R}}_+^n$ such that

$$\begin{aligned} \sum _{i=1}^n\Vert x_i(t)-x_{i\infty }\Vert _{L^p(\Omega )} \leqslant C({\varvec{x}}^0,{\varvec{x}}_\infty )e^{-\lambda t/(2p)}, \quad t>0,\ p\geqslant 1, \end{aligned}$$

where ${\varvec{x}}^0={\varvec{x}}(0)$ and the constant $\lambda >0$ is constructive up to a finite-dimensional inequality. Before we make this result precise, we review the state of the art and explain the main difficulties and key ideas.

### State of the Art

The research of the large-time asymptotics of general reaction–diffusion systems with diagonal diffusion, modeling chemical reactions has experienced dramatic scientific progress in recent years. One reason for this progress is due to new developments of so-called entropy methods. Classical methods include linearized stability techniques, spectral theory, invariant region arguments, and Lyapunov stability; see, e.g., [15, 26]. The entropy method is a genuinely nonlinear approach without using any kind of linearization; it is rather robust against model variations, and it is able to provide explicitly computable decay rates. The first related works date back to the 1980s [29, 30]. The obtained results are restricted to two space dimensions and do not provide explicit estimates, since the proofs are based on contradiction arguments. First applications of the entropy method that provide explicit rates and constants were concerned with particular cases, like two-component systems [17], four-component systems [19], or multicomponent linear systems [20]. Later, nonlinear reaction networks with an arbitrary number of chemical substances were considered [24, 43]. Exponential convergence of close-to-equilibrium solutions to quadratic reaction–diffusion systems with detailed balance was shown in [10]. reaction–diffusion systems without detailed balance [23] and with complex balance [18, 44, 53] were also thoroughly investigated. The convergence to equilibrium was proven for rather general solution concepts, like very weak solutions [46] and renormalized solutions [25].

The large-time behavior of solutions to cross-diffusion systems is less studied. The convergence to equilibrium was shown for the Shigesada–Kawasaki–Teramoto population model with Lotka–Volterra terms in [50, 55] without any rate and in [11] without reaction terms. The exponential decay of solutions to volume-filling population systems, again without reaction terms, was proved in [58].

A number of articles are concerned with the large-time asymptotics in Maxwell–Stefan systems. For global existence results on these systems, we refer to [31, 40, 41]. In [40], the exponential decay to the homogeneous state is shown with vanishing reaction rates and same molar masses. The result was generalized to different molar masses in [12], but still without reaction terms. The convergence to equilibrium was proved in [27, Theorem 9.7.4] and [31, Theorem 4.3] under the condition that the initial datum is close to the equilibrium state. The work [31] also addresses the exponential convergence to a homogeneous equilibrium assuming (i) global existence of strong solutions and (ii) uniform-in-time strict positivity of the solutions (see Prop. 4.4 therein). A similar result, but for two-phase systems, was proved in [7]. The novelty in our paper is that we also provide a global existence proof (which avoids assumption (i)) and that we replace the strong assumption (ii) by a natural condition on the reactions, namely that there exist no equilibria on $\partial {\mathbb {R}}_+^n$. We note that there exists a large class of chemical reaction networks, called *concordant networks*, which possess no boundary equilibria [51, Theorem 2.8(ii)].

We finally remark that the mathematical study of the Maxwell–Stefan diffusion system is a dynamic field, and many works have been carried out after the submission of our paper. We refer the interested reader to the incomplete list [2, 3, 14, 34, 39, 47, 48] of recent works.

### Key Ideas

The analysis of the Maxwell–Stefan equations (1) is rather delicate. The first difficulty is that the fluxes are not given as linear combinations of the gradients of the mass fractions, which makes it necessary to invert the flux-gradient relations in (1). However, summing the equations for $\nabla x_i$ in (1) for $i=1,\ldots ,n$, we see that the Maxwell–Stefan equations are linear dependent, and we need to invert them on a subspace [5]. The idea is to work with the $n-1$ variables ${\varvec{\rho }}'=(\rho _1,\ldots ,\rho _{n-1})^\top$ by setting $\rho _n=1-\sum _{i=1}^{n-1}\rho _i$, i.e., the mass density of the last component (often the solvent) is computed from the other mass densities. Then there exists a diffusion matrix $\mathbb {A}({\varvec{\rho }}')\in {\mathbb {R}}^{(n-1)\times (n-1)}$ such that system (1) can be written as

7 $$\begin{aligned} \partial _t{\varvec{\rho }}' - {\text {div}}(\mathbb {A}({\varvec{\rho }}')\nabla {\varvec{x}}') = {\varvec{r}}'({\varvec{x}}), \end{aligned}$$

where ${\varvec{x}}'=(x_1,\ldots ,x_{n-1})^\top$ and ${\varvec{r}}'=(r_1,\ldots ,r_{n-1})^\top$. The matrix $\mathbb {A}({\varvec{\rho }}')$ is generally neither symmetric nor positive definite. However, equations (7) exhibit a formal gradient-flow structure [40]. This means that we introduce the so-called (relative) entropy density

8 $$\begin{aligned} h({\varvec{\rho }}') = c\sum _{i=1}^n x_i\ln \frac{x_i}{x_{i\infty }}, \quad \text{ where } \rho _n=1-\sum _{i=1}^{n-1} \rho _i, \end{aligned}$$

and the entropy variable ${\varvec{w}}=(w_1,\ldots ,w_{n-1})^\top$ with $w_i=\partial h/\partial \rho _i$. Here, ${\varvec{x}}_\infty \in \mathcal {E}$ is an arbitrary detailed-balance equilibrium. We associate to the entropy density the relative entropy (or free energy)

9 $$\begin{aligned} E[{\varvec{x}}|{\varvec{x}}_\infty ] = \int _\Omega h({\varvec{\rho }}')\hbox {d}z = \sum _{i=1}^n\int _\Omega cx_i\ln \frac{x_i}{x_{i\infty }}\hbox {d}z. \end{aligned}$$

Denoting by $h''({\varvec{\rho }}')$ the Hessian of *h* with respect to ${\varvec{\rho }}'$, equation (7) is equivalent to

10 $$\begin{aligned} \partial _t{\varvec{\rho }}' - {\text {div}}(\mathbb {B}({\varvec{w}})\nabla {\varvec{w}}) = {\varvec{r}}'({\varvec{x}}), \end{aligned}$$

where $\mathbb {B}({\varvec{w}})=\mathbb {A}({\varvec{\rho }}')h''({\varvec{\rho }}')^{-1}$ is symmetric and positive definite [12, Lemma 10 (iv)] and ${\varvec{\rho }}'$ and ${\varvec{x}}$ are functions of ${\varvec{w}}$. The elliptic operator can be formulated as $\mathbb {K}{\text {grad}}h({\varvec{\rho }}')$, where $\mathbb {K}\xi ={\text {div}}(\mathbb {B}\nabla \xi )$ is the Onsager operator and grad is the functional derivative. This formulation motivates the notion “gradient-flow structure”.

The second difficulty comes from the fact that the cross-diffusion coupling prevents the use of standard tools like maximum principles and regularity theory. In particular, it is not clear how to prove lower and upper bounds for the mass densities or molar fractions. Surprisingly, this problem can be also solved by the transformation to entropy variables. Indeed, the mapping $(0,1)^{n-1}\rightarrow {\mathbb {R}}^{n-1}$, ${\varvec{\rho }}'\mapsto {\varvec{w}}$, can be inverted, and the image ${\varvec{\rho }}'({\varvec{w}})$ lies in $(0,1)^{n-1}$ and satisfies $1-\sum _{i=1}^{n-1}\rho _i<1$. If all molar masses are equal, $M=M_i$, the inverse function can be written explicitly as $\rho _i({\varvec{w}})=\exp (Mw_i)(1+\sum _{j=1}^{n-1}\exp (Mw_j))^{-1}$; for the general case, see Lemma 5 below. This yields the positivity and $L^\infty$ bounds for $\rho _i$ without the use of a maximum principle. To make this argument rigorous, we first need to solve (10) for ${\varvec{w}}$ and then to conclude that ${\varvec{\rho }}'={\varvec{\rho }}'({\varvec{w}})$ solves (1).

Summarizing, the entropy helps us to “symmetrize” system (1) and to derive $L^\infty$ bounds. There is a further benefit: the entropy is a Lyapunov functional along solutions to the detailed-balance system (1). Indeed, a formal computation shows the following relation (a weaker discrete version is made rigorous in the proof of Theorem 4):

11 $$\begin{aligned} \frac{\hbox {d}}{\hbox {d}t}E[{\varvec{x}}|{\varvec{x}}_\infty ] + D[{\varvec{x}}] = 0,\quad t>0, \end{aligned}$$

where the entropy production

12 $$\begin{aligned} D[{\varvec{x}}] = \sum _{i,j=1}^{n-1}\int _\Omega B_{ij}({\varvec{w}})\nabla w_i\cdot \nabla w_j \hbox {d}z + \sum _{a=1}^N\int _\Omega (k_f^a{\varvec{x}}^{{\varvec{\alpha }}^a}-k_b^a{\varvec{x}}^{{\varvec{\beta }}^a}) \ln \frac{k_f^a {\varvec{x}}^{{\varvec{\alpha }}^a}}{k_b^a {\varvec{x}}^{{\varvec{\beta }}^a}}\hbox {d}z \end{aligned}$$

is nonnegative (due to Lemmas 6 and 7). Here, $B_{ij}$ are the coefficients of the matrix $\mathbb {B}$. Exponential decay follows if the entropy entropy-production inequality

13 $$\begin{aligned} D[{\varvec{x}}] \geqslant \lambda E[{\varvec{x}}|{\varvec{x}}_\infty ] \end{aligned}$$

holds for all suitable functions ${\varvec{x}}$ and for some $\lambda >0$. Note that this functional inequality does not hold for all detailed-balance equilibria, but only for those who satisfy certain conservation laws. The existence and uniqueness of such equilibria is proved in Theorem 11. Inserting inequality (13) into (11) yields

$$\begin{aligned} \frac{\hbox {d}}{\hbox {d}t}E[{\varvec{x}}|{\varvec{x}}_\infty ] + \lambda E[{\varvec{x}}|{\varvec{x}}_\infty ] \leqslant 0, \quad t>0, \end{aligned}$$

and Gronwall’s inequality allows us to conclude that

$$\begin{aligned} E[{\varvec{x}}(t)|{\varvec{x}}_\infty ] \leqslant E[{\varvec{x}}(0)|{\varvec{x}}_\infty ] e^{-\lambda t}, \quad t>0. \end{aligned}$$

By a variant of the Csiszár–Kullback–Pinsker inequality (Lemma 18), this gives exponential decay in the $L^1$ norm with rate $\lambda /2$ and, by interpolation, in the $L^p$ norm with rate $\lambda /(2p)$ for all $1\leqslant p<\infty$. An important feature of this result is that the constant $\lambda$ is constructive up to a finite-dimensional inequality.

The cornerstone of the convergence to equilibrium is to prove inequality (13). In comparison to previous results for reaction–diffusion systems, e.g. [24, 43], the difference here is that the reactions are defined in terms of molar fractions, while the conservation laws are written in terms of concentrations. This difference causes the main difficulty in proving (13), except in very special cases, e.g., when all molar masses are equal (in this case, the molar fraction and concentration are proportional) or in case of equal homogeneities (see Section 3.4). Naturally, one could express the molar fractions by the concentrations, i.e. $x_i = c_i/(\sum _{i=1}^{n}c_i)$, but this extremely complicates the formulation of the entropy production $D[{\varvec{x}}]$, which in turn makes the analysis of (13) inaccessible. The key idea here is to introduce the total concentration $c = \sum _{i=1}^{n}c_i$ as an independent variable and to rewrite $D[{\varvec{x}}]$ in terms of $x_i = c_i/c$. This, in combination with an estimate for $E[{\varvec{x}}|{\varvec{x}}_\infty ]$ in terms of $c_i$ and *c*, allows us to adapt the ideas from previous works on reaction–diffusion systems to finally obtain the desired inequality (13).

### Main Results

Our main result is the exponential convergence to equilibrium. For this, we need to show some intermediate results. The existence of solutions to (1), (3) was shown in [12] without reaction terms. Therefore, we prove the global existence of bounded weak solutions to (1), (3) with reaction terms (2). The proof follows that one in [12] but the estimates related to the reaction terms are different. A key step is the proof of the monotonicity of ${\varvec{w}}\mapsto \sum _{i=1}^{n-1} r_i({\varvec{x}})$; see Lemma 7.

Second, we derive the conservation laws satisfied by the solutions to (1) (Lemma 9) and prove the existence of a positive detailed-balance equilibrium ${\varvec{x}}_\infty$ satisfying (5) and the conservation laws (Theorem 11). The existence of unique equilibrium states for chemical reaction networks is well studied in the literature (see, e.g., [21]), but not in the present framework. One difficulty is the additional constraint $\sum _{i=1}^n x_i=1$, which significantly complicates the analysis. The key idea for the existence of a unique detailed-balance equilibrium is to analyze systems in the partial concentrations $c_1,\ldots ,c_n$*and* the total concentration *c*, considered as an independent variable. The increase of the dimension of the system from *n* to $n+1$ allows us to apply geometric arguments and a result of Feinberg [21] to achieve the claim.

Third, we prove the entropy entropy-production inequality (13) (Prop. 19 and 26). The proof follows basically from [25, Lemma 2.7] when the stoichiometric coefficients satisfy $\sum _{i=1}^n\alpha _i^a=\sum _{i=1}^n\beta _i^a$ for all $a=1,\ldots ,N$, since this property allows us to replace the molar fractions $x_i$ by the concentrations $c_i$. If the property is not fulfilled, we work again in the augmented space of concentrations $(c_1,\ldots ,c_n,c)$. One step of the proof (Lemma 22) requires the proof of an inequality whose constant is constructive only up to a finite-dimensional inequality. We believe that for concrete systems, this constant can be computed in a constructive way. We present such an example in Section 4.

Before stating the main theorem, we need some notation. Let

$$\begin{aligned} \mathbb {W} = ({\varvec{\beta }}^a-{\varvec{\alpha }}^a)_{a=1,\ldots ,N}\in {\mathbb {R}}^{n\times N}, \end{aligned}$$

be the Wegscheider matrix (or stoichiometric coefficients matrix) and set $$m=\dim {\text {ker}}(\mathbb {W}^\top )$$$$>0$$. We choose a matrix $${\mathbb {Q}}\in {\mathbb {R}}^{m\times n}$$ whose rows form a basis of $${\text {ker}}(\mathbb {W}^\top )$$. Let $${{\varvec{M}}^0}\in {\mathbb {R}}_+^m$$ be the initial mass vector, which depends on $${\varvec{c}}^0$$ (see Lemma 9) and let $${\varvec{\zeta }}\in {\mathbb {R}}^{1\times m}$$ be a row vector satisfying $${\varvec{\zeta }}{\mathbb {Q}}=(M_1,\ldots ,M_n)$$ and $${\varvec{\zeta }}{{\varvec{M}}^0}=1$$. We show in Lemma 10 that such a vector $${\varvec{\zeta }}$$ always exists. Its appearance comes from the constraint $$\sum _{i=1}^n x_i=1$$; such a vector is not needed in reaction–diffusion systems like in [25]. Given $${{\varvec{M}}^0}\in {\mathbb {R}}_+^m$$ such that $${\varvec{\zeta }}{{\varvec{M}}^0}=1$$, we prove in Section 3.2 that there exists a unique positive *detailed-balance equilibrium*$${\varvec{x}}_\infty \in \mathcal {E}$$ satisfying

14 $$\begin{aligned} {\mathbb {Q}}{\varvec{c}}_\infty = {{\varvec{M}}^0}, \quad \sum _{i=1}^n x_{i\infty }=1, \end{aligned}$$

where the components of ${\varvec{c}}_\infty$ are given by $c_{i\infty }=x_{i\infty }/\sum _{i=1}^n M_ix_{i\infty }$. The first expression in (14) are the conservation laws, while the second one is the normalization condition.

Note that besides the unique positive detailed-balance equilibrium (for a fixed initial mass vector), there could exist possibly infinitely many *boundary equilibria*, i.e. ${\varvec{x}}^*\in \partial \mathcal {E}$ such that ${\varvec{x}}^*$ solves (14). We need to exclude such equilibria. For a discussion of boundary equilibria and the Global Attractor Conjecture, we refer to Remark 15.

1. Data: $\Omega \subset {\mathbb {R}}^d$ with $d\geqslant 1$ is a bounded domain with Lipschitz boundary, $T>0$, and $D_{ij}=D_{ji}>0$ for $i,j=1,\ldots ,n$, $i\ne j$.
2. Detailed-balance condition: $\mathcal {E}\ne \emptyset$, where $\mathcal {E}$ is defined in (6).
3. Initial condition: ${\varvec{\rho }}^0\in L^1(\Omega ;{\mathbb {R}}^n)$ with $\rho _i^0\geqslant 0$, $\sum _{i=1}^n\rho _i^0=1$, and the initial entropy is finite, $\int _\Omega h({{\varvec{\rho }}^0}')\hbox {d}z<\infty$, where *h* is defined in (8) with some ${\varvec{x}}_\infty \in \mathcal {E}$.

The main result is as follows:

#### Theorem 1

(Convergence to equilibrium) Let Assumptions (A1)–(A3) hold. Let ${{\varvec{M}}^0}\in {\mathbb {R}}_+^m$ be a positive initial mass vector satisfying ${\varvec{\zeta }} {{\varvec{M}}^0}= 1$. Then

(i) There exists a global bounded weak solution ${\varvec{\rho }}=(\rho _1,\ldots ,\rho _n)^\top$ to (1)–(2) in the sense of Theorem 4 below;
(ii) There exists a unique ${\varvec{x}}_\infty \in \mathcal {E}$ satisfying (14), where the set of equilibria $\mathcal {E}$ is defined in (6);
(iii) Assume in addition that the system (1)–(2) has no boundary equilibria. Then there exist constants $C>0$ and $\lambda >0$, which are constructive up to a finite-dimensional inequality, such that, if ${\varvec{\rho }}^0$ satisfies additionally $\mathbb Q\int _{\Omega }{\varvec{c}}^0\hbox {d}z = {{\varvec{M}}^0}$, the following exponential convergence to equilibrium holds:
  $$\begin{aligned} \sum _{i=1}^n\Vert x_i(t)-x_{i\infty }\Vert _{L^p(\Omega )} \leqslant Ce^{-\lambda t/(2p)} \big (E[{\varvec{x}}^0|{\varvec{x}}_\infty ]\big )^{1/(2p)} ,\quad t>0, \end{aligned}$$
  where $1\leqslant p <\infty$, $x_i=\rho _i/(cM_i)$ with $c=\sum _{i=1}^n\rho _i/M_i$, $E[{\varvec{x}}|{\varvec{x}}_\infty ]$ is the relative entropy defined in (9), ${\varvec{\rho }}$ is the solution constructed in (i), and ${\varvec{x}}_\infty$ is constructed in (ii).

#### Remark 2

(Classical and weak solustions) Theorem 1(i) provides the global existence of a weak solution with physical initial data, while the local existence of a classical solution, with more regular initial data, was already proved in [5], based on general results on normally elliptic operators. Both the global existence of a classical solution as well as the uniqueness of weak solutions for Maxwell–Stefan reaction-cross-diffusion systems are extremely difficult to prove. On the other hand, a weak-strong uniqueness result might be achievable; see [13] for such a result for a different class of reaction-cross-diffusion systems. We leave this interesting open question to future investigations.

#### Remark 3

(Complex balance)] We show in Theorem 11 that system (1) with the reaction terms (2) possesses a unique positive detailed-balance equilibrium. This means that we have assumed the reversibility of the reaction system. This assumption is rather strong, and it is well known in chemical reaction network theory that it can be significantly generalized to complex-balance systems. Here, the balance is not assumed to hold for any elementary reaction step but only for the total in-flow and total out-flow of each chemical complex. We are able to extend our results to this situation as well, considering the reaction terms (54); see Theorem 33 in Section 5.

Clearly, any detailed-balance equilibrium is also a complex-balance equilibrium, and Theorem 1 is included in Theorem 33. However, to make the proofs as accessible as possible, we prefer to present the detailed-balance case in full detail and sketch the extension to complex-balance systems. $\quad \square$

The paper is organized as follows: Part (i) of Theorem 1 is proved in Section 2. In Section 3, the conservation laws are derived, the existence of a detailed-balance equilibrium and the entropy entropy-production inequality (13) are proved, and the convergence result is shown. Section 4 is concerned with a specific example for which the constant in the entropy entropy-production inequality can be computed explicitly. The results are extended to complex-balance systems in Section 5. Finally, we prove the technical Lemma 21 in the appendix.

### Notation

We use the following notation:

- Bold letters indicate vectors in ${\mathbb {R}}^n$ (e.g. ${\varvec{c}}=(c_1,\ldots ,c_n)^\top$).
- Normal letters denote the sum of all the components of the corresponding letter in bold font (e.g. $c=\sum _{i=1}^n c_i$).
- Primed bold letters signify that the last component is removed from the original vector (e.g. ${\varvec{c}}'=(c_1,\ldots ,c_{n-1})^\top$).
- Overlined letters usually denote integration over $\Omega$ (e.g. $\overline{{\varvec{c}}}=\int _\Omega {\varvec{c}}\hbox {d}z$ or $\overline{c_i}=\int _\Omega c_i\hbox {d}z$).
- If $f:{\mathbb {R}}\rightarrow {\mathbb {R}}$ is a function and ${\varvec{c}}\in {\mathbb {R}}^n$ a vector, the expression $f({\varvec{c}})$ denotes the vector $(f(c_1),\ldots ,f(c_n))^\top$.
- Let ${\varvec{x}}$, ${\varvec{\alpha }}\in (0,\infty )^n$. The expression ${\varvec{x}}^{{\varvec{\alpha }}}$ equals the product $\prod _{i=1}^n x_i^{\alpha _i}$.
- Matrices are generally denoted by double-barred capital letters (e.g. $\mathbb {A}\in {\mathbb {R}}^{m\times n}$).

The inner product in ${\mathbb {R}}^n$ is denoted by $\langle \cdot ,\cdot \rangle$, $|\Omega |$ is the measure of $\Omega$, and we set ${\mathbb {R}}_+=(0,\infty )$. In the estimates, $C>0$ denotes a generic constant with values changing from line to line.

## Global Existence of Weak Solutions

We prove part (i) of Theorem 1. Throughout this section, we fix an arbitrary detailed-balance equilibrium ${\varvec{x}}_\infty \in \mathcal {E}$. Due to (A2), such a vector ${\varvec{x}}_\infty$ always exists. The existence result is stated more precisely in the following theorem:

### Theorem 4

(Global existence) Let Assumptions (A1)–(A3) hold. Then there exists a bounded weak solution ${\varvec{\rho }}=(\rho _1,\ldots ,\rho _n)^\top$ to (1)–(3) satisfying $\rho _i\geqslant 0$, $\sum _{i=1}^n\rho _i=1$ in $\Omega \times (0,T)$ and

$$\begin{aligned} \rho _i\in L^2(0,T;H^1(\Omega )),\ \partial _t\rho _i\in L^2(0,T;H^1(\Omega )'), \quad i=1,\ldots ,n, \end{aligned}$$

i.e., for all $$q_1,\ldots ,q_{n-1}\in L^2(0,T;H^1(\Omega ))$$,

15 $$\begin{aligned} \sum _{i=1}^{n-1}\int _0^T\langle \partial _t\rho _i,q_i\rangle \mathrm{d}t + \sum _{i,j=1}^{n-1}\int _0^T\int _\Omega A_{ij}({\varvec{\rho }}')\nabla x_i\cdot \nabla q_j \mathrm{d}z\mathrm{d}t = \sum _{i=1}^{n-1}\int _0^T\int _\Omega r_i({\varvec{x}})q_i \mathrm{d}z\mathrm{d}t, \end{aligned}$$

where ${\varvec{x}}=(x_1,\ldots ,x_n)^\top$, $x_i=\rho _i/(cM_i)$ for $i=1,\ldots ,n-1$, $x_n=1-\sum _{i=1}^{n-1}x_i$, $c=\sum _{i=1}^n\rho _i/M_i$, and $\mathbb {A}=(A_{ij})$ is the diffusion matrix in (7).

The proof is similar to the one given in [12]. Since in that paper no reaction terms have been considered, we need to show how these terms can be controlled. First, we collect some results.

### Preliminary Results

A straightforward computation (see [12, Lemma 5]) shows that the entropy variables are given by

16 $$\begin{aligned} w_i = \frac{\partial h}{\partial \rho _i} = \frac{1}{M_i}\ln \frac{x_i}{x_{i\infty }} - \frac{1}{M_n}\ln \frac{x_n}{x_{n\infty }}, \quad i=1,\ldots ,n-1, \end{aligned}$$

recalling *h* defined in (8). Given ${\varvec{\rho }}'=(\rho _1,\ldots ,\rho _{n-1})^\top$, this formula and the relation $x_i=\rho _i/(cM_i)$ allow us to compute ${\varvec{w}}=(w_1,\ldots ,w_{n-1})^\top$. The following lemma states that the mapping ${\varvec{\rho }}'\mapsto {\varvec{w}}$ can be inverted:

#### Lemma 5

Let $${\varvec{w}}=(w_1,\ldots ,w_{n-1})^\top \in {\mathbb {R}}^{n-1}$$ be given. Then there exists a unique vector $${\varvec{\rho }}'=(\rho _1,\ldots ,$$$$\rho _{n-1})^\top \in (0,1)^{n-1}$$ satisfying $$\sum _{i=1}^{n-1}\rho _i<1$$ such that (16) holds with $$\rho _n=1-\sum _{i=1}^{n-1}\rho _i>0$$, $$x_i=\rho _i/(cM_i)$$ and $$c=\sum _{i=1}^n\rho _i/M_i$$. Moreover, the function $${\varvec{\rho }}':{\mathbb {R}}^{n-1}\rightarrow (0,1)^{n-1}$$, $$(w_1,\ldots ,w_{n-1})^\top \mapsto {\varvec{\rho }}'(w)=(\rho _1,\ldots ,\rho _{n-1})^\top $$ is bounded.

#### Proof

First, we show that there exists a unique vector $(x_1,\ldots ,x_{n-1})^\top \in (0,1)^{n-1}$ satisfying (16) with $x_n=1-\sum _{i=1}^{n-1}x_i>0$ (see [12, Lemma 6]). Let $z_i:=x_{i\infty }/x_{n\infty }^{M_i/M_n}$. The function

$$\begin{aligned} f(s)=\sum _{i=1}^{n-1}z_i(1-s)^{M_i/M_n}\exp (M_iw_i) \end{aligned}$$

is strictly decreasing in [0, 1] and $0=f(1)<f(s)<f(0)=\sum _{i=1}^{n-1}\exp (M_iw_i)z_i$. Thus, there exists a unique fixed point $s_0\in (0,1)$ such that $f(s_0)=s_0$. Defining $x_i=z_i(1-s_0)^{M_i/M_n}\exp (M_iw_i)$ for $i=1,\ldots ,n-1$, we infer that $x_i>0$, $\sum _{i=1}^{n-1}x_i=f(s_0)=s_0<1$, and (16) holds with $x_n:=1-s_0$.

Next, let $(x_1,\ldots ,x_{n-1})^\top \in (0,1)^{n-1}$ and $x_n:=1-\sum _{i=1}^{n-1}x_i>0$ be given and define $\rho _i=cM_ix_i$, where $c=1/(\sum _{i=1}^{n}M_ix_i)$. Then $(\rho _1,\ldots ,\rho _{n-1})^\top \in (0,1)^{n-1}$ is the unique vector satisfying $\rho _n=1-\sum _{i=1}^{n-1}\rho _i>0$, $x_i=\rho _i/(cM_i)$ for $i=1,\ldots ,n-1$, and $c=\sum _{i=1}^n\rho _i/M_i$ [12, Lemma 7]. Finally, the result follows by combining the previous steps. $\quad \square$

#### Lemma 6

Let ${\varvec{w}}\in H^1(\Omega ;{\mathbb {R}}^{n-1})$. Then there exists a constant $C_B>0$, which only depends on $D_{ij}$ and $M_i$, such that

$$\begin{aligned} \int _\Omega \nabla {\varvec{w}}:\mathbb {B}({\varvec{w}})\nabla {\varvec{w}} \mathrm{d}z \geqslant C_B\sum _{i=1}^n\int _\Omega |\nabla x_i^{1/2}|^2 \mathrm{d}z, \end{aligned}$$

where “:” means summation over both matrix indices.

We recall that $\mathbb {B}(w)=\mathbb {A}({\varvec{\rho }}')h''({\varvec{\rho }}')^{-1}$ and $h''$ is the Hessian of the entropy *h* defined in (8). Lemma 6 is proved in [12, Lemma 12]. It is shown in [12, Lemma 9] that $\mathbb {B}$ is symmetric and positive definite.

### Solution to an Approximate Problem

Let $T>0$, $M\in {\mathbb {N}}$, $\tau =T/M$, $k\in \{1,\ldots ,M\}$, $\varepsilon >0$, and $l\in {\mathbb {N}}$ with $l>d/2$. Then the embedding $H^l(\Omega )\hookrightarrow L^\infty (\Omega )$ is compact. Given ${\varvec{w}}^{k-1}\in L^\infty (\Omega ;{\mathbb {R}}^{n-1})$, we wish to find ${\varvec{w}}^k\in H^l(\Omega ;{\mathbb {R}}^{n-1})$ such that

17 $$\begin{aligned} \frac{1}{\tau }\int _\Omega&\big ({\varvec{\rho }}'({\varvec{w}}^k)-{\varvec{\rho }}'({\varvec{w}}^{k-1})\big ) \cdot {\varvec{q}}\hbox {d}z + \int _\Omega \nabla {\varvec{q}}:\mathbb {B}({\varvec{w}}^k)\nabla {\varvec{w}}^k \hbox {d}z \nonumber \\&{}+ \varepsilon \int _\Omega \bigg (\sum _{|{\varvec{\alpha }}|=l}D^{{\varvec{\alpha }}} {\varvec{w}}^k :D^{{\varvec{\alpha }}} {\varvec{q}} + {\varvec{w}}^k\cdot {\varvec{q}}\bigg )\hbox {d}z = \int _\Omega {\varvec{r}}'({\varvec{x}}^k)\cdot {\varvec{q}}\hbox {d}z \end{aligned}$$

for all $${\varvec{q}}\in H^l(\Omega ;{\mathbb {R}}^{n-1})$$, where $${\varvec{r}}'=(r_1,\ldots ,r_{n-1})^\top $$, $$x^k_i=\rho _i({\varvec{w}}^k)/(cM_i)$$, and $${\varvec{\rho }}'({\varvec{w}}^k)$$ is defined in Lemma 5. Moreover, $${\varvec{\alpha }}=(\alpha _1,\ldots ,\alpha _d)\in {\mathbb {N}}_0^d$$ is a multi-index of order $$|{\varvec{\alpha }}|=\alpha _1+\cdots +\alpha _d=l$$ and $$D^{{\varvec{\alpha }}}=\partial ^{|{\varvec{\alpha }}|}/(\partial z_1^{\alpha _1} \cdots $$$$\partial z_d^{\alpha _d})$$ is a partial derivative of order *l*. The regularization with the *l*th-order derivative terms is needed since the matrix $$\mathbb {B}$$ is not uniformly positive definite. As $${\varvec{\rho }}'$$ is a bounded function of $${\varvec{w}}$$, we can apply the boundedness-by-entropy method of [37] or [12, Section 3.1] to deduce the existence of a weak solution $${\varvec{w}}^k\in H^l(\Omega ;{\mathbb {R}}^{n-1})$$ to (17).

### Uniform Estimates

The crucial step is to derive some a priori estimates. The idea is to employ the test function ${\varvec{q}}={\varvec{w}}^k$ in (17) and to proceed as in the proof of Lemma 14 of [12]. The reaction terms have no influence, as the following lemma shows:

#### Lemma 7

It holds that

$$\begin{aligned} {\varvec{r}}'({\varvec{x}}^k)\cdot {\varvec{w}}^k = \sum _{i=1}^{n-1} r_i({\varvec{x}}^k)w_i^k\leqslant 0. \end{aligned}$$

#### Proof

Let ${\varvec{x}}={\varvec{x}}^k$ and ${\varvec{w}}={\varvec{w}}^k$ to simplify. We deduce from (16) and total mass conservation (4) that $\sum _{i=1}^{n-1}r_i({\varvec{x}})=-r_n({\varvec{x}})$ and

18 $$\begin{aligned} {\varvec{r}}'({\varvec{x}})\cdot {\varvec{w}}&= \sum _{i=1}^{n-1}r_i({\varvec{x}})\bigg (\frac{1}{M_i}\ln \frac{x_i}{x_{i\infty }} - \frac{1}{M_n}\ln \frac{x_n}{x_{n\infty }}\bigg ) \nonumber \\&= \sum _{i=1}^{n-1}\frac{r_i({\varvec{x}})}{M_i}\ln \frac{x_i}{x_{i\infty }} - \frac{1}{M_n}\ln \frac{x_n}{x_{n\infty }}\sum _{i=1}^{n-1}r_i({\varvec{x}}) = \sum _{i=1}^{n}\frac{r_i({\varvec{x}})}{M_i}\ln \frac{x_i}{x_{i\infty }}. \end{aligned}$$

In view of definition (2) of $r_i$ and ${\varvec{x}}_\infty \in \mathcal {E}$, the last expression becomes

$$\begin{aligned} {\varvec{r}}'({\varvec{x}})\cdot {\varvec{w}}&= \sum _{i=1}^n\sum _{a=1}^N(\beta _i^a-\alpha _i^a) (k_f^a {\varvec{x}}^{{\varvec{\alpha }}^a}-k_b^a {\varvec{x}}^{{\varvec{\beta }}^a})\ln \frac{x_i}{x_{i\infty }} \\&= \sum _{i=1}^n\sum _{a=1}^N (k_f^a {\varvec{x}}^{{\varvec{\alpha }}^a}-k_b^a {\varvec{x}}^{{\varvec{\beta }}^a}) \ln \frac{x_i^{\beta _i^a}x_{i\infty }^{\alpha _i^a}}{x_i^{\alpha _i^a} x_{i\infty }^{\beta _i^a}} \\&= \sum _{a=1}^N (k_f^a {\varvec{x}}^{{\varvec{\alpha }}^a}-k_b^a {\varvec{x}}^{{\varvec{\beta }}^a}) \ln \frac{{\varvec{x}}^{{\varvec{\beta }}^a}{\varvec{x}}_{\infty }^{{\varvec{\alpha }}^a}}{{\varvec{x}}^{{\varvec{\alpha }}^a} {\varvec{x}}_{\infty }^{{\varvec{\beta }}^a}} \\&= \sum _{a=1}^N (k_f^a {\varvec{x}}^{{\varvec{\alpha }}^a}-k_b^a {\varvec{x}}^{{\varvec{\beta }}^a}) \ln \frac{k_b^a {\varvec{x}}^{{\varvec{\beta }}^a}}{k_f^a {\varvec{x}}^{{\varvec{\alpha }}^a}} \leqslant 0, \end{aligned}$$

because of the monotonicity of the logarithm. $\quad \square$

Taking into account Lemma 7, the estimations of Section 3.2 in [12] lead to the discrete entropy inequality

19 $$\begin{aligned} \int _\Omega h(({\varvec{\rho }}')^k)\hbox {d}z&+ C\tau \sum _{j=1}^k\sum _{i=1}^{n}\Vert \nabla (x_i^j)^{1/2}\Vert _{L^2(\Omega )}^2 + \tau \sum _{j=1}^k\sum _{i=1}^n\int _\Omega (-r_i({\varvec{x}}^j)\cdot {\varvec{w}}^j) \hbox {d}z \nonumber \\&+ \varepsilon \tau \sum _{j=1}^k\sum _{i=1}^{n-1}\int _\Omega \bigg (\sum _{|\alpha |=l}(D^{{\varvec{\alpha }}} w_i^j)^2 + (w_i^j)^2\bigg )\hbox {d}z \leqslant \int _\Omega h(({\varvec{\rho }}')^0_\eta )\hbox {d}z, \end{aligned}$$

where $({\varvec{\rho }}')^0_\eta$ is the vector of strictly positive approximations of the initial vector $({\varvec{\rho }}^0)'=(\rho _1^0,\ldots ,\rho _{n-1}^0)^\top$ and $C>0$ is a generic constant independent of $\tau$ and $\varepsilon$. This shows that

$$\begin{aligned} \tau \sum _{j=1}^k\Vert x_i^j\Vert _{H^1(\Omega )}^2 + \varepsilon \tau \sum _{j=1}^n\Vert w_i^j\Vert _{H^l(\Omega )}^2 \leqslant C, \quad i=1,\ldots ,n, \end{aligned}$$

where $C>0$ is independent of $\varepsilon$ and $\tau$. From these estimates and the boundedness of the reaction terms, we infer a uniform bound for the discrete time derivative:

$$\begin{aligned} \tau \sum _{k=1}^M\sum _{i=1}^{n-1} \big \Vert \tau ^{-1}(\rho _i^k-\rho _i^{k-1})\big \Vert _{H^l(\Omega )'}^2 \leqslant C. \end{aligned}$$

These estimates are sufficient to perform the limit $\varepsilon \rightarrow 0$ and $\tau \rightarrow 0$ in (17) as in Section 3.3 of [12] showing that the limit satisfies (15) and therefore is a global weak solution to (1)–(2).

#### Remark 8

(Discrete entropy inequality) Before summing from $j=1,\ldots ,k$, we can formulate the discrete entropy inequality (19) as

$$\begin{aligned} E[{\varvec{x}}^k|{\varvec{x}}_\infty ] + \tau D[{\varvec{x}}^k] + C\varepsilon \tau \sum _{i=1}^{n-1}\Vert w_i^k\Vert _{H^l(\Omega )}^2 \leqslant E[{\varvec{x}}^{k-1}|{\varvec{x}}_\infty ]. \end{aligned}$$

This estimate is the discrete analogue of (11) and it will be needed in the proof of part (iii) of Theorem 1; see Section 3.6. $\quad \square$

## Convergence to Equilibrium Under Detailed Balance

In this section, we prove parts (ii) and (iii) of Theorem 1. First, we discuss the conservation laws and the existence of an equilibrium state.

### Conservation Laws

We set $R_i=r_i/M_i$, ${\varvec{J}}_i={\varvec{j}}_i/M_i$ and ${\varvec{R}} = (R_1,\ldots ,R_n)^\top$, $\mathbb {J}=({\varvec{J}}_1,\ldots ,{\varvec{J}}_n)^\top$, ${\varvec{c}}=(c_1,\ldots ,c_n)^\top$, where we recall that $c_i=\rho _i/M_i$. Dividing the *i*th-equation of (1) by $M_i$, we can reformulate them in vector form as

20 $$\begin{aligned} \partial _t{\varvec{c}} + {\text {div}}\mathbb {J} = {\varvec{R}}. \end{aligned}$$

Let $\mathbb {W}=(\beta _i^a-\alpha _i^a)\in {\mathbb {R}}^{n\times N}$ be the Wegscheider matrix and let $m=\dim {\text {ker}}(\mathbb {W}^\top )$. Note that $m\geqslant 1$ since it follows from the conservation of total mass, $\sum _{i=1}^n r_i({\varvec{x}})=0$, that ${\varvec{M}}^\top \mathbb {W}=0$, i.e., the vector ${\varvec{M}}=(M_1,\ldots ,M_n)^\top$ belongs to ${\text {ker}}(\mathbb {W}^\top )$. Let the row vectors ${\varvec{q}}_1,\ldots ,{\varvec{q}}_m\in {\mathbb {R}}^{1\times n}$ be a basis of the left null space of $\mathbb {W}$, i.e. ${\varvec{q}}_i\mathbb {W}=0$ for $i=1,\ldots ,m$. In particular, ${\varvec{q}}_i^\top \in {\text {ker}}(\mathbb {W}^\top )$. Finally, let ${\mathbb {Q}}=(Q_{ij})\in {\mathbb {R}}^{m\times n}$ be the matrix with rows ${\varvec{q}}_j$.

We claim that system (20) (with no-flux boundary conditions) possesses precisely *m* linear independent conservation laws.

#### Lemma 9

(Conservation laws) Let ${\varvec{\rho }}$ be a weak solution to (1)–(2) in the sense of Theorem 4. Then the following conservation laws hold:

$$\begin{aligned} {\mathbb {Q}}\overline{{\varvec{c}}}(t) = {{\varvec{M}}^0}, \quad t>0, \end{aligned}$$

where ${{\varvec{M}}^0}={\mathbb {Q}}\overline{{\varvec{c}}}^0$ is called the initial mass vector and $c_i^0=\rho _i^0/M_i$, $i=1,\ldots ,n$.

Note that, by changing the sign of the rows of ${\mathbb {Q}}$ if necessary, we can always choose ${\mathbb {Q}}$ such that ${{\varvec{M}}^0}$ is positive componentwise.

#### Proof

We observe that the definitions of $${\mathbb {Q}}$$ and $$r_i({\varvec{x}}) = M_iR_i({\varvec{x}})$$ in (2) imply that $${\mathbb {Q}}{\varvec{R}}=0$$. Choosing $${\varvec{q}}_j=(Q_{j1},\ldots ,$$$$Q_{jn})$$ as a test function in the weak formulation of (20) and observing that $$\nabla {\varvec{q}}_j=0$$, we find that

$$\begin{aligned} \int _0^t\int _\Omega \partial _t({\mathbb {Q}}{\varvec{c}})_j\hbox {d}z \hbox {d}s= & {} \sum _{i=1}^n\int _0^t\int _\Omega \partial _t c_i Q_{ji}\hbox {d}z \hbox {d}s = \sum _{i=1}^n\int _0^t\int _\Omega R_iQ_{ji}\hbox {d}z\hbox {d}s \\= & {} \int _0^t\int _\Omega ({\mathbb {Q}}{\varvec{R}})_j\hbox {d}z\hbox {d}s = 0. \end{aligned}$$

This shows that

$$\begin{aligned} \int _\Omega {\mathbb {Q}}{\varvec{c}}(t)\hbox {d}z = \int _\Omega {\mathbb {Q}}{\varvec{c}}^0\hbox {d}z, \quad t>0, \end{aligned}$$

or ${\mathbb {Q}}\overline{{\varvec{c}}}(t)={\mathbb {Q}}\overline{{\varvec{c}}}^0=:{{\varvec{M}}^0}$, where $c_i^0=\rho _i^0/M_i$ is the initial concentration. $\quad \square$

#### Lemma 10

There exists a row vector ${\varvec{\zeta }}\in {\mathbb {R}}^{1\times m}$ such that ${\varvec{\zeta }}{\mathbb {Q}}={\varvec{M}}^\top$ and ${\varvec{\zeta }}{{\varvec{M}}^0}=1$.

#### Proof

Since ${\varvec{M}}$ lies in the kernel of $\mathbb {W^\top }$ and the rows of ${\mathbb {Q}}$ form a basis of this space, we have ${\varvec{M}}\in {\text {ker}}(\mathbb {W}^\top )={\text {ran}}({\mathbb {Q}}^\top )$. We infer that there exists a row vector ${\varvec{\zeta }}\in {\mathbb {R}}^{1\times m}$ such that ${\mathbb {Q}}^\top {\varvec{\zeta }}^\top ={\varvec{M}}$ or ${\varvec{\zeta }}{\mathbb {Q}}={\varvec{M}}^\top$. Moreover, by recalling $|\Omega | = 1$ and $\sum _{i=1}^n\rho _i^0 = 1$ in $\Omega$,

$$\begin{aligned} 1 = \int _\Omega \sum _{i=1}^{n}\rho _i^0\hbox {d}z = \sum _{i=1}^n\overline{\rho _i}^0 = \sum _{i=1}^n M_i\overline{c_i}^0 = {\varvec{M}}^\top \overline{{\varvec{c}}}^0 = {\varvec{\zeta }}{\mathbb {Q}}\overline{{\varvec{c}}}^0 = {\varvec{\zeta }}{{\varvec{M}}^0}, \end{aligned}$$

using the definition of ${{\varvec{M}}^0}$ in Lemma 9. $\quad \square$

### Detailed-Balance Condition

The relative entropy (9) is formally a Lyapunov functional along the trajectories of (1)–(2) for ${\varvec{x}}_\infty \in \mathcal {E}$. Note that $\mathcal {E}$ generally is a manifold of detailed-balance equilibria. To identify uniquely the detailed-balance equilibrium, we need to take into account the conservation laws. This subsection is concerned with the existence of a unique positive detailed-balance equilibrium satisfying the conservation laws.

For chemical reaction networks in the context of ordinary differential equations (ODE), the existence of a unique equilibrium state was proved by Horn and Jackson [33]; also see [21]. The difficulty in this work lies in the fact that the reactions are modeled by molar fractions ${\varvec{x}}$, while the conservation laws are presented by concentrations ${\varvec{c}}$. Our idea is to enlarge the space ${\mathbb {R}}_+^n$ of concentrations $(c_1,\ldots ,c_n)$ by adding the total concentration $c = \sum _{i=1}^{n}c_i \in {\mathbb {R}}_+$, which is considered to be an independent variable, and then to employ the ideas by Feinberg [21] to the augmented space ${\mathbb {R}}_+^{n+1}$. To this end, let

21 $$\begin{aligned} {\varvec{\omega }} = (\omega _1,\ldots ,\omega _{n+1}) = (c_1,\ldots ,c_n,c), \end{aligned}$$

and define the vectors in ${\mathbb {R}}^{n+1}$

22 $$\begin{aligned} \begin{aligned} {\varvec{\mu }}^a&= \bigg (\alpha _1^a,\ldots ,\alpha _n^a, \bigg (\sum _{i=1}^n(\beta _i^a-\alpha _i^a)\bigg )^+\bigg ), \\ {\varvec{\nu }}^a&= \bigg (\beta _1^a,\ldots ,\beta _n^a, \bigg (\sum _{i=1}^n(\alpha _i^a-\beta _i^a)\bigg )^+\bigg ), \end{aligned} \end{aligned}$$

where $y^+=\max \{0,y\}$. Finally, we write $\mathbf {1}_n=(1,\ldots ,1)^\top \in {\mathbb {R}}^n$ and $\mathbf {1}_{n+1}=(1,\ldots ,1)^\top \in {\mathbb {R}}^{n+1}$. The main result of this subsection is the following:

#### Theorem 11

(Existence of a unique detailed-balance equilibrium) Assume that (A2) holds and let ${{\varvec{M}}^0}\in {\mathbb {R}}_+^m$ be an initial mass vector and ${\varvec{\zeta }}\in {\mathbb {R}}^{1\times m}$ be a row vector such that ${\varvec{\zeta }}{{\varvec{M}}^0}=1$. Then there exists a unique positive detailed-balance equilibrium ${\varvec{x}}_\infty \in \mathcal {E}$ satisfying the conservation laws and the normalization condition (14).

To prove Theorem 11 we first show the existence of an “equilibrium” in the augmented space.

#### Proposition 12

Suppose the assumptions of Theorem 11 hold. Then there exists a unique ${\varvec{\omega }}\in {\mathbb {R}}_+^{n+1}$ satisfying

23 $$\begin{aligned} k_f^a{\varvec{\omega }}^{{\varvec{\mu }}^a} = k_b^a{\varvec{\omega }}^{{\varvec{\nu }}^a}, \quad a=1,\ldots ,N, \quad \widehat{\mathbb {Q}}{\varvec{\omega }} = {\widehat{{\varvec{M}}}}^0, \end{aligned}$$

where $\widehat{{\mathbb {Q}}}$ and ${\widehat{{\varvec{M}}}}^0$ are defined by

$$\begin{aligned} \widehat{{\mathbb {Q}}} = \begin{pmatrix} {\mathbb {Q}}&{} {\varvec{0}} \\ {\varvec{1}}_n^\top &{} -1 \end{pmatrix}\in {\mathbb {R}}^{(m+1)\times (n+1)}, \quad {\widehat{{\varvec{M}}}}^0= \begin{pmatrix} {{\varvec{M}}^0}\\ 0 \end{pmatrix}\in {\mathbb {R}}^{n+1}. \end{aligned}$$

Before proving this result, we first show that Theorem 11 follows from Proposition 12.

#### Proof of Theorem 11

Let ${\varvec{\omega }} = (c_{1\infty }, \ldots , c_{n\infty }, c_\infty )$ be the equilibrium in the augmented space constructed in Proposition 12. Define $x_{i\infty }=c_{i\infty }/c_\infty$. We will prove that ${\varvec{x}}_\infty$ is an element of $\mathcal {E}$ and satisfies (14). Indeed, for any $a = 1,\ldots , N$, let $\gamma ^a:=\sum _{i=}^n(\alpha _i^a-\beta _i^a)$ and assume first that $\gamma ^a\geqslant 0$. Then

$$\begin{aligned} k_f^a\prod _{i=1}^n c_{i\infty }^{\alpha _i^a} = k_f^a{\varvec{\omega }}^{{\varvec{\mu }}^a} = k_b^a{\varvec{\omega }}^{{\varvec{\nu }}^a} = k_b^a\prod _{i=1}^n c_{i\infty }^{\beta _i^a}c_\infty ^{\gamma ^a} \end{aligned}$$

is equivalent to

$$\begin{aligned} k_f^a{\varvec{x}}_\infty ^{{\varvec{\alpha }}^a} = k_f^a\prod _{i=1}^n c_{i\infty }^{\alpha _i^a} c_\infty ^{-\sum _{i=1}^n\alpha _i^a} = k_b^a\prod _{i=1}^n c_{i\infty }^{\beta _i^a} c_\infty ^{-\sum _{i=1}^n\beta _i^a} = k_b^a{\varvec{x}}_\infty ^{{\varvec{\beta }}^a}. \end{aligned}$$

The case $\gamma ^a\leqslant 0$ can be treated in an analogous way. Thus, ${\varvec{x}}_\infty \in \mathcal {E}$. It follows immediately from $\widehat{{\mathbb {Q}}}{\varvec{\omega }} = \widehat{{\varvec{M}}}^0$ that ${\mathbb {Q}}{\varvec{c}}_\infty = {{\varvec{M}}^0}$ and $\sum _{i=1}^{n}c_{i\infty } = c_\infty$. The latter identity implies that $\sum _{i=1}^n x_{i\infty } = 1$ due to $x_{i\infty } = c_{i\infty }/c_\infty$. Therefore ${\varvec{x}}_\infty$ satisfies (14). $\quad \square$

The aim now is to prove Proposition 12. For this, we introduce the following definitions:

$$\begin{aligned} X_1&= \bigg \{{\varvec{\omega }}\in {\mathbb {R}}_+^{n+1}:\;k_f^a{\varvec{\omega }}^{{\varvec{\mu }}^a} = k_b^a{\varvec{\omega }}^{{\varvec{\nu }}^a} \text{ for } a=1,\ldots ,N\bigg \}, \\ X_2&= \bigg \{{\varvec{\omega }}\in {\mathbb {R}}_+^{n+1}:\; \widehat{{\mathbb {Q}}}{\varvec{\omega }}={\widehat{{\varvec{M}}}}^0\bigg \}. \end{aligned}$$

We argue that $X_1$ and $X_2$ are not empty. Indeed, due to (A2), there exists ${\varvec{x}}_\infty \in \mathcal {E}$. Fix any $\omega _{n+1,\infty }\in (0,\infty )$ and define $\omega _{i\infty } = x_{i\infty }\omega _{n+1,\infty }$ for all $i=1,\ldots , n$. We obtain immediately ${\varvec{\omega }}_\infty = (\omega _{1\infty },\ldots , \omega _{n+1,\infty })\in X_1$. Concerning $X_2$, we see that there exists ${\varvec{\omega }}' = (\omega _1,\ldots , \omega _n) \in {\mathbb {R}}_+^n$ such that ${\mathbb {Q}}{\varvec{\omega }}' = {{\varvec{M}}^0}$ since $\mathrm {rank}({\mathbb {Q}}) = m < n$. By defining $\omega _{n+1} = \sum _{i=1}^n\omega _i$, we infer that ${\varvec{\omega }} = ({\varvec{\omega }}', \omega _{n+1})\in X_2$.

#### Lemma 13

Let ${{\varvec{M}}^0}\in {\mathbb {R}}_+^m$ and ${\varvec{\zeta }}\in {\mathbb {R}}^{1\times m}$ with ${\varvec{\zeta }}{{\varvec{M}}^0}=1$, let ${\varvec{\omega }}_\infty \in X_1$ and ${\varvec{p}}\in X_2$. Then the following statements are equivalent:

- There exists a unique vector ${\varvec{\omega }}\in X_1\cap X_2$.
- There exists a unique vector ${\varvec{\varphi }}^*\in {\text {span}}\{{\varvec{q}}_1^\top ,\ldots ,{\varvec{q}}_m^\top \}$ (${\varvec{q}}_i$ is the *i*th row of ${\mathbb {Q}}$) and a unique number $z_{m+1}\in {\mathbb {R}}$ such that
  24 $$\begin{aligned} {\varvec{\omega }}'_\infty e^{{\varvec{\varphi }}^*} - e^{-z_{m+1}}{\varvec{p}}'\in {\text {ker}}{\mathbb {Q}}, \quad \langle e^{{\varvec{\varphi }}^*}{\varvec{\omega }}'_\infty ,\mathbf {1}_n\rangle = \omega _{n+1,\infty }. \end{aligned}$$

Here, we denote ${\varvec{p}}'=(p_1,\ldots ,p_{n})$ and ${\varvec{\omega }}'_\infty e^{{\varvec{\varphi }}^*}$ equals the vector with components $\omega _{i\infty } e^{\varphi _i^*}$, $i=1,\ldots ,n$. Observe that ${\text {span}}\{{\varvec{q}}_1^\top ,\ldots ,{\varvec{q}}_m^\top \} ={\text {ran}}({\mathbb {Q}}^\top )$.

#### Proof

We first claim that

$$\begin{aligned} X_1&= \bigg \{{\varvec{\omega }}\in {\mathbb {R}}_+^{n+1}:\;\exists z_{m+1}\in {\mathbb {R}},\ {\varvec{\varphi }}^*\in {\text {ran}}({\mathbb {Q}}^\top )\; \text { such that }\; {\varvec{\omega }} = e^{z_{m+1}}\begin{pmatrix} {\varvec{\omega }}'_\infty e^{{\varvec{\varphi }}^*} \\ \omega _{n+1,\infty } \end{pmatrix}\bigg \}. \end{aligned}$$

Indeed, ${\varvec{\omega }}\in X_1$ holds if and only if ${\varvec{\omega }}_\infty ^{{\varvec{\nu }}^a-{\varvec{\mu }}^a}=k_f^a/k_b^a ={\varvec{\omega }}^{{\varvec{\nu }}^a-{\varvec{\mu }}^a}$. Taking the logarithm componentwise, this becomes

$$\begin{aligned} \langle \log {\varvec{\omega }}_\infty ,{\varvec{\nu }}^a-{\varvec{\mu }}^a\rangle = \langle \log {\varvec{\omega }},{\varvec{\nu }}^a-{\varvec{\mu }}^a\rangle , \quad a=1,\ldots ,N. \end{aligned}$$

This means that ${\varvec{\varphi }}:=\log ({\varvec{\omega }}/{\varvec{\omega }}_\infty ) = \log {\varvec{\omega }}-\log {\varvec{\omega }}_\infty \in {\text {ker}} \{{\varvec{\nu }}^a-{\varvec{\mu }}^a\}_{a=1,\ldots ,N}$. By definition of ${\varvec{\mu }}^a$ and ${\varvec{\nu }}^a$, we know that

$$\begin{aligned} {\text {ker}}\{{\varvec{\nu }}^a-{\varvec{\mu }}^a\}_{a=1,\ldots ,N} = {\text {span}}\big \{({\varvec{q}}_1^\top ,0)^\top ,\ldots ,({\varvec{q}}_m^\top ,0)^\top , \mathbf {1}_{n+1}\big \}. \end{aligned}$$

Thus, there exist numbers $z_1,\ldots ,z_{m+1}\in {\mathbb {R}}$ such that

$$\begin{aligned} {\varvec{\varphi }} = \sum _{i=1}^m z_i \begin{pmatrix} {\varvec{q}}_i^\top \\ 0 \end{pmatrix} + z_{m+1}\mathbf {1}_{n+1} = \begin{pmatrix}{\varvec{\varphi }}^* + z_{m+1}\mathbf {1}_n \\ z_{m+1} \end{pmatrix}, \end{aligned}$$

where ${\varvec{\varphi }}^*=\sum _{i=1}^m z_i{\varvec{q}}_i^\top \in \mathrm{ran}({\mathbb {Q}}^\top )$. It follows from the definition of ${\varvec{\varphi }}$ that

$$\begin{aligned} \frac{{\varvec{\omega }}}{{\varvec{\omega }}_\infty } = e^{{\varvec{\varphi }}} = \exp \begin{pmatrix}{\varvec{\varphi }}^* + z_{m+1}\mathbf {1}_n \\ z_{m+1} \end{pmatrix} = e^{z_{m+1}}\begin{pmatrix} e^{{\varvec{\varphi }}^*} \\ 1 \end{pmatrix}. \end{aligned}$$

We conclude that ${\varvec{\omega }}\in X_1$ if and only if

$$\begin{aligned} {\varvec{\omega }} = {\varvec{\omega }}_\infty e^{z_{m+1}} \begin{pmatrix} e^{{\varvec{\varphi }}^*} \\ 1 \end{pmatrix} = e^{z_{m+1}}\begin{pmatrix} {\varvec{\omega }}'_\infty e^{{\varvec{\varphi }}^*} \\ \omega _{n+1,\infty } \end{pmatrix}, \end{aligned}$$

and this proves the claim.

Next, fixing ${\varvec{p}}\in X_2$, it holds that ${\varvec{\omega }}\in X_2$ if and only if

$$\begin{aligned} {\varvec{0}}&= \widehat{{\mathbb {Q}}}({\varvec{\omega }}-{\varvec{p}}) = \begin{pmatrix} {\mathbb {Q}}&{} {\varvec{0}} \\ {\varvec{1}}_n^\top &{} -1 \end{pmatrix} \begin{pmatrix} {\varvec{\omega }}'-{\varvec{p}}' \\ \omega _{n+1}-p_{n+1} \end{pmatrix} \\&= \begin{pmatrix} {\mathbb {Q}}({\varvec{\omega }}'-{\varvec{p}}') \\ \langle \mathbf {1}_n,{\varvec{\omega }}'-{\varvec{p}}'\rangle - (\omega _{n+1}-p_{n+1}) \end{pmatrix}. \end{aligned}$$

Consequently, in view of the preceding claim, we have ${\varvec{\omega }}\in X_1\cap X_2$ if and only if

$$\begin{aligned} {\varvec{0}} = \widehat{\mathbb {Q}}({\varvec{\omega }}-{\varvec{p}}) = \begin{pmatrix} {\mathbb {Q}}(e^{z_{m+1}}{\varvec{\omega }}'_\infty e^{{\varvec{\varphi }}^*}-{\varvec{p}}') \\ \langle \mathbf {1}_n,e^{z_{m+1}}{\varvec{\omega }}'_\infty e^{{\varvec{\varphi }}^*}-{\varvec{p}}'\rangle - (e^{z_{m+1}}\omega _{n+1,\infty }-p_{n+1}) \end{pmatrix}. \end{aligned}$$

The first *n* rows mean that ${\varvec{\omega }}'_\infty e^{{\varvec{\varphi }}*}-e^{-z_{m+1}}{\varvec{p}}'\in {\text {ker}}{\mathbb {Q}}$. Since ${\varvec{p}}\in X_2$ and consequently $p_{n+1}=\sum _{i=1}^n p_i =\langle \mathbf {1}_n,{\varvec{p}}'\rangle$, the last row simplifies to

$$\begin{aligned} 0 = e^{z_{m+1}}\big (\langle e^{{\varvec{\varphi }}^*}{\varvec{\omega }}'_\infty ,\mathbf {1}_n\rangle - \omega _{n+1,\infty }\big ). \end{aligned}$$

This shows (24) and ends the proof. $\quad \square$

We need one more lemma.

#### Lemma 14

[21, Proposition B.1] Let *U* be a linear subspace of ${\mathbb {R}}^n$ and $a=(a_1,\ldots ,a_n), b=(b_1,\ldots ,b_n) \in {\mathbb {R}}_+^n$. There exists a unique element $\mu =(\mu _1,\ldots , \mu _n) \in U^{\perp }$ such that

$$\begin{aligned} ae^{\mu } - b \in U, \end{aligned}$$

where $$ae^\mu =(a_1 e^{\mu _1},\ldots ,a_n e^{\mu _n})$$.

#### Proof of Proposition 12

*Step 1: Existence.* First, fixing ${\varvec{\omega }}_\infty \in X_1$ and ${\varvec{p}}\in X_2$, we claim that there exist $z_{m+1}\in {\mathbb {R}}$ and ${\varvec{\varphi }}^*\in {\text {ran}}({\mathbb {Q}}^\top )$ such that (24) holds. We apply Lemma 14 with $U={\text {ker}}{\mathbb {Q}}$, $a={\varvec{\omega }}'_\infty$, and $b=e^{-z_{m+1}}{\varvec{p}}'$, yielding the existence of a unique vector ${\varvec{\varphi }}^*(z_{m+1})\in U^\perp ={\text {ran}}({\mathbb {Q}}^\top )$ such that

25 $$\begin{aligned} {\varvec{\omega }}'_\infty e^{{\varvec{\varphi }}^*(z_{m+1})} - e^{-z_{m+1}}{\varvec{p}}'\in {\text {ker}}{\mathbb {Q}}. \end{aligned}$$

It remains to show the second equation in (24), i.e. to show that there exists a number $z_{m+1}^*\in {\mathbb {R}}$ such that $\langle e^{{\varvec{\varphi }}^*(z^*_{m+1})}{\varvec{\omega }}'_\infty ,\mathbf {1}_n\rangle =\omega _{n+1,\infty }$. Then we set ${\varvec{\varphi }}^*:={\varvec{\varphi }}^*(z_{m+1}^*)$, and (25) yields the first equation in (24).

We know that ${\varvec{M}}\in {\text {span}}\{{\varvec{q}}_1^\top ,\ldots ,{\varvec{q}}_m^\top \}$. Then (25) implies that

$$\begin{aligned} \big \langle {\varvec{\omega }}'_\infty e^{{\varvec{\varphi }}^*(z_{m+1})} - e^{-z_{m+1}}{\varvec{p}}',{\varvec{M}}\big \rangle = 0 \quad \text{ or }\quad \big \langle {\varvec{\omega }}'_\infty e^{{\varvec{\varphi }}^*(z_{m+1})},{\varvec{M}}\big \rangle = e^{-z_{m+1}}\langle {\varvec{p}}',{\varvec{M}}\rangle > 0. \end{aligned}$$

We deduce that

$$\begin{aligned} \lim _{z_{m+1}\rightarrow +\infty }\langle {\varvec{\omega }}'_\infty e^{{\varvec{\varphi }}^*(z_{m+1})}, {\varvec{M}}\rangle = 0, \quad \lim _{z_{m+1}\rightarrow -\infty }\langle {\varvec{\omega }}'_\infty e^{{\varvec{\varphi }}^*(z_{m+1})}, {\varvec{M}}\rangle = \infty . \end{aligned}$$

Moreover, since

$$\begin{aligned} \frac{1}{M_{\mathrm{max}}}\langle {\varvec{\omega }}'_\infty e^{{\varvec{\varphi }}^*(z_{m+1})}, {\varvec{M}}\rangle \leqslant \langle {\varvec{\omega }}'_\infty e^{{\varvec{\varphi }}^*(z_{m+1})},\mathbf {1}_n\rangle \leqslant \frac{1}{M_\mathrm{min}}\langle {\varvec{\omega }}'_\infty e^{{\varvec{\varphi }}^*(z_{m+1})}, {\varvec{M}}\rangle , \end{aligned}$$

it holds that

$$\begin{aligned} \lim _{z_{m+1}\rightarrow +\infty }\langle {\varvec{\omega }}'_\infty e^{{\varvec{\varphi }}^*(z_{m+1})}, \mathbf {1}_n\rangle = 0, \quad \lim _{z_{m+1}\rightarrow -\infty }\langle {\varvec{\omega }}'_\infty e^{{\varvec{\varphi }}^*(z_{m+1})}, \mathbf {1}_n\rangle = \infty . \end{aligned}$$

By continuity, there exists $z_{m+1}^*\in {\mathbb {R}}$ such that $\langle e^{{\varvec{\varphi }}^*(z^*_{m+1})}{\varvec{\omega }}'_\infty ,\mathbf {1}_n\rangle =\omega _{n+1,\infty }$.

*Step 2: Uniqueness.* Assume that there exist $(\widehat{{\varvec{\varphi }}},\widehat{z})$ and  with $\widehat{{\varvec{\varphi }}}$,  and $\widehat{z}$,  such that2627From (26) it follows thatWe infer from  thatHence, we have $I_2=-I_1$ and because ofit holds that $I_2=-I_1\leqslant 0$.

Now, if , Lemma 14 shows that , and the proof is finished. Thus, let us assume, without loss of generality, that . Then the definition and nonpositivity of $I_2$ imply that28Consider the function $f:{\mathbb {R}}^n\rightarrow {\mathbb {R}}$, $f({\varvec{\varphi }})=\sum _{i=1}^n \omega _{i\infty } e^{\varphi _i}$. Then $\mathrm {D}f({\varvec{\varphi }})={\varvec{\omega }}'_\infty e^{{\varvec{\varphi }}}$ and $\mathrm {D}^2f({\varvec{\varphi }})={\text {diag}}(\omega _{i\infty } e^{\varphi _i})_{i=1,\ldots ,n}$ and so, *f* is strictly convex. Hence, by (27),We deduce from this identity and (28) that  and consequently, $I_2=0$ and $I_1=-I_2=0$. By the monotonicity of the exponential function, we infer that . Then, taking the difference of the two vectors in (26), we have . Since , this shows that ${\varvec{p}}'\in {\text {ker}}{\mathbb {Q}}$ and therefore ${\mathbb {Q}}{\varvec{p}}'={\varvec{0}}$ contradicting the fact that ${\varvec{p}}\in X_2$ and in particular ${\mathbb {Q}}{\varvec{p}}'={{\varvec{M}}^0}\ne {\varvec{0}}$. Thus, $\widehat{z}$ and  must coincide, and uniqueness holds. $\quad \square$

#### Remark 15

(Boundary equilibria and Global Attractor Conjecture) Besides the unique positive detailed-balance equilibrium obtained in Theorem 11, there might exist (possibly infinitely many) boundary equilibria ${\varvec{x}}^*\in \partial \mathcal {E}$. The convergence of solutions to reaction systems towards the positive equilibrium under the presence of boundary equilibria is a subtle problem, even in the ODE setting. The main reason for there is that if a trajectory converges to a boundary equilibrium, the entropy production $D[{\varvec{x}}]$ vanishes while the relative entropy $E[{\varvec{x}}|{\varvec{x}}_\infty ]$ remains positive, which means that the entropy-production inequality (13) is not true in general. However, it is conjectured, still in the ODE setting, that the positive detailed-balance equilibrium is the only attracting point despite the presence of boundary equilibria. This is called the *Global Attractor Conjecture*, and it is considered as one of the most important problems in chemical reaction network theory; see, e.g., [1, 28] for partial answers. Recently, a full proof of this conjecture in the ODE setting has been proposed in [16], but the result is still under verification; see also [18, 25] for reaction–diffusion systems possessing boundary equilibria. $\quad \square$

### Preliminary Estimates for the Entropy and Entropy Production

We derive some estimates for the relative entropy (9) and the entropy production (12) from below and above. In what follows, let $\rho _1,\ldots ,\rho _n:\Omega \rightarrow [0,\infty )$ be integrable functions such that $\sum _{i=1}^n\rho _i=1$ in $\Omega$ and set $c_i=\rho _i/M_i$ and $x_i=c_i/c$ for $i=1,\ldots ,n$. We assume that the functions have the same regularity as the weak solutions from Theorem 4. For later reference, we note the following inequalities, which give bounds on the total concentration only depending on the molar masses:

29 $$\begin{aligned} \frac{1}{M_{\mathrm{max}}} \leqslant c = \sum _{i=1}^n\frac{\rho _i}{M_i} \leqslant \frac{1}{M_\mathrm{min}} \quad \text{ in } \Omega , \end{aligned}$$

where $M_{\mathrm{max}}=\max _{i=1,\ldots ,n}M_i$ and $M_\mathrm{min}=\min _{i=1,\ldots ,n}M_i$. Moreover, given the unique equilibrium ${\varvec{x}}_\infty$ according to Theorem 11, we observe that $\sum _{i=1}^n\rho _{i\infty }/M_i=\sum _{i=1}^n c_{i\infty } =c_\infty \sum _{i=1}^n x_{i\infty }=c_\infty$, and consequently,

30 $$\begin{aligned} \frac{1}{M_{\mathrm{max}}} \leqslant c_\infty \leqslant \frac{1}{M_\mathrm{min}}. \end{aligned}$$

#### Lemma 16

There exists a constant $C>0$, only depending on $M_\mathrm{min}$, $M_{\mathrm{max}}$, and ${\varvec{x}}_\infty$, such that

$$\begin{aligned} E[{\varvec{x}}|{\varvec{x}}_\infty ] \leqslant C\sum _{i=1}^n\bigg (\int _\Omega \Big (c_i^{1/2}-\overline{c_i^{1/2}}\,\Big )^2 \hbox {d}z + \big (\overline{c_i}^{1/2}-c_{i\infty }^{1/2}\big )^2\bigg ). \end{aligned}$$

#### Proof

We use $\sum _{i=1}^n x_i=\sum _{i=1}^n x_{i\infty }=1$ to reformulate the relative entropy

$$\begin{aligned} E[{\varvec{x}}|{\varvec{x}}_\infty ]&= \sum _{i=1}^n\int _\Omega c\bigg (x_i\ln \frac{x_i}{x_{i\infty }} - x_i + x_{i\infty } \bigg )\hbox {d}z \\&= \sum _{i=1}^n\int _\Omega cx_{i\infty } \bigg (\frac{x_i}{x_{i\infty }}\ln \frac{x_i}{x_{i\infty }} - \frac{x_i}{x_{i\infty }} + 1\bigg )\hbox {d}z. \end{aligned}$$

The function $\Phi (y)=(y\ln y-y+1)/(y^{1/2}-1)^2$ is continuous and nondecreasing on ${\mathbb {R}}_+$. Therefore, using (29),

31 $$\begin{aligned} E[{\varvec{x}}|{\varvec{x}}_\infty ]&= \sum _{i=1}^n\int _\Omega cx_{i\infty } \Phi \bigg (\frac{x_i}{x_{i\infty }}\bigg )\bigg (\bigg (\frac{x_i}{x_{i\infty }}\bigg )^{1/2} -1\bigg )^2 \hbox {d}z \nonumber \\&\leqslant \frac{1}{M_\mathrm{min}}\sum _{i=1}^n\Phi \bigg (\frac{1}{x_{i\infty }}\bigg )\frac{1}{x_{i\infty }} \int _\Omega (x_i-x_{i\infty })^2 \hbox {d}z \leqslant C\sum _{i=1}^n\int _\Omega (x_i-x_{i\infty })^2 \hbox {d}z \end{aligned}$$

for some constant $C>0$ only depending on $M_\mathrm{min}$ and ${\varvec{x}}_\infty$.

It remains to formulate the square on the right-hand side in terms of the partial concentrations. To this end, we set $f_i({\varvec{c}}) = c_i/c$ for ${\varvec{c}}=(c_1,\ldots ,c_n)$ and $c=\sum _{j=1}^n c_j$. By definition of the molar fractions $x_i$ and $x_{i\infty }$, we have $x_i=f_i({\varvec{c}})$ and $x_{i\infty }=f_i({\varvec{c}}_\infty )$. The estimates

$$\begin{aligned} \left| \frac{\partial f_i}{\partial c_j}({\varvec{c}})\right| \leqslant \frac{1}{c} \leqslant M_{\mathrm{max}}, \quad \left| \frac{\partial f_i}{\partial c_j}({\varvec{c}}_\infty )\right| \leqslant \frac{1}{c_\infty } \leqslant M_{\mathrm{max}} \end{aligned}$$

imply that, for some ${\varvec{\xi }}$ on the line between ${\varvec{c}}$ and ${\varvec{c}}_\infty$,

$$\begin{aligned} \int _\Omega (x_i-x_{i\infty })^2 \hbox {d}z&= \int _\Omega (f_i({\varvec{c}})-f_i({\varvec{c}}_\infty ))^2\hbox {d}z = \sum _{j=1}^n\int _\Omega \bigg (\frac{\partial f_i}{\partial c_j}({\varvec{\xi }})\bigg )^2 (c_j-c_{j\infty })^2 \hbox {d}z \\&\leqslant M_{\mathrm{max}}^2\sum _{j=1}^n\int _\Omega \big (c_j^{1/2}+c_{j\infty }^{1/2}\big )^2\big (c_j^{1/2}-c_{j\infty }^{1/2}\big )^2 \hbox {d}z \\&\leqslant M_{\mathrm{max}}^2\bigg (\frac{2}{M_\mathrm{min}^{1/2}}\bigg )^2\sum _{i=1}^n\int _\Omega \big (c_i^{1/2}-c_{i\infty }^{1/2}\big )^2 \hbox {d}z \\&\leqslant C\sum _{i=1}^n\int _\Omega \big (c_i^{1/2}-c_{i\infty }^{1/2}\big )^2 \hbox {d}z, \end{aligned}$$

and $C>0$ depends only on $M_\mathrm{min}$, $M_{\mathrm{max}}$, and ${\varvec{x}}_\infty$. Combining this estimate with (31) leads to (here, we use that $|\Omega |=1$)

32 $$\begin{aligned} E[{\varvec{x}}|{\varvec{x}}_\infty ]&\leqslant C\sum _{i=1}^n\int _\Omega \big (c_i^{1/2}-c_{i\infty }^{1/2}\big )^2 \hbox {d}z \nonumber \\&\leqslant 2C\sum _{i=1}^n\bigg (\int _\Omega \Big (c_i^{1/2}-\overline{c_{i}^{1/2}}\,\Big )^2 \hbox {d}z + \Big (\,\overline{c_{i}^{1/2}}-c_{i\infty }^{1/2}\Big )^2\bigg ) \nonumber \\&\leqslant 2C\sum _{i=1}^n\bigg (\int _\Omega \Big (c_i^{1/2}-\overline{c_{i}^{1/2}}\,\Big )^2 \hbox {d}z + 2\Big (\,\overline{c_{i}^{1/2}}-\overline{c_i}^{1/2}\Big )^2 + 2\big (\overline{c_i}^{1/2}-c_{i\infty }^{1/2}\big )^2\bigg ). \end{aligned}$$

We wish to estimate the second term. The Cauchy–Schwarz inequality gives that $\overline{c_i^{1/2}}\leqslant \overline{c_i}^{1/2}$, and hence

$$\begin{aligned} \Big (\,\overline{c_{i}^{1/2}}-\overline{c_i}^{1/2}\Big )^2&= \Big (\overline{c_{i}^{1/2}}\Big )^2 + \overline{c_i} - 2\overline{c_{i}^{1/2}}\overline{c_i}^{1/2} \\&\leqslant \Big (\overline{c_{i}^{1/2}}\Big )^2 + \overline{c_i} - 2\overline{c_{i}^{1/2}}\,\overline{c_i^{1/2}} = \int _\Omega \Big (c_i^{1/2}-\overline{c_i^{1/2}}\,\Big )^2 \hbox {d}z. \end{aligned}$$

Putting this into (32), it follows that

$$\begin{aligned} E[{\varvec{x}}|{\varvec{x}}_\infty ] \leqslant 2C\sum _{i=1}^n\bigg (3\int _\Omega \Big (c_i^{1/2}-\overline{c_{i}^{1/2}}\,\Big )^2 \hbox {d}z + 2\big (\overline{c_i}^{1/2}-c_{i\infty }^{1/2}\big )^2\bigg ), \end{aligned}$$

and we conclude the proof. $\quad \square$

#### Lemma 17

There exists a constant $C>0$, only depending on $M_\mathrm{min}$ and $M_{\mathrm{max}}$, such that

$$\begin{aligned} D[{\varvec{x}}] \geqslant C\left[ \sum _{i=1}^n\int _\Omega |\nabla c_i^{1/2}|^2 \hbox {d}z + \int _\Omega |\nabla c^{1/2}|^2 \hbox {d}z + \sum _{a=1}^N\int _\Omega \big (k_f^a{\varvec{x}}^{{\varvec{\alpha }}^a}-k_b^a{\varvec{x}}^{{\varvec{\beta }}^a}\big ) \ln \frac{k_f^a{\varvec{x}}^{{\varvec{\alpha }}^a}}{k_b^a{\varvec{x}}^{{\varvec{\beta }}^a}}\hbox {d}z\right] . \end{aligned}$$

#### Proof

Lemma 6 shows that the first term in $D[{\varvec{x}}]$ can be estimated from below:

$$\begin{aligned} \int _\Omega \nabla {\varvec{w}}:\mathbb {B}({\varvec{w}})\nabla {\varvec{w}} \hbox {d}z \geqslant C_B\sum _{i=1}^n\int _\Omega |\nabla x_i^{1/2}|^2 \hbox {d}z. \end{aligned}$$

We claim that we can relate $\sum _{i=1}^n|\nabla x_i^{1/2}|^2$ and $|\nabla c^{1/2}|^2$. For this, we proceed as in [12, p. 494]. We infer from the definition $x_i=c_i/c$ that $c\sum _{i=1}^n M_ix_i=\sum _{i=1}^n M_ic_i=\sum _{i=1}^n\rho _i=1$. Therefore, inserting $c=1/\sum _{i=1}^n M_ix_i$ and using the Cauchy–Schwarz inequality,

33 $$\begin{aligned} |\nabla c^{1/2}|^2&= \frac{1}{4c}|\nabla c|^2 = \frac{1}{4c}\bigg |\frac{-\sum _{i=1}^nM_i\nabla x_i}{(\sum _{i=1}^n M_ix_i)^2}\bigg |^2 = c^3\bigg |\sum _{i=1}^n M_i x_i^{1/2}\nabla x_i^{1/2}\bigg |^2 \nonumber \\&\leqslant nc^3\sum _{i=1}^n M_i^2 x_i|\nabla x_i^{1/2}|^2 \leqslant \frac{nM_{\mathrm{max}}^2}{M_\mathrm{min}^3}\sum _{i=1}^n|\nabla x_i^{1/2}|^2, \end{aligned}$$

where we used $c\leqslant 1/M_\mathrm{min}$ (see (29)). Similarly, employing (33),

34 $$\begin{aligned} \sum _{i=1}^n|\nabla c_i^{1/2}|^2&= \sum _{i=1}^n|\nabla (cx_i)^{1/2}|^2 \leqslant 2\sum _{i=1}^n x_i|\nabla c^{1/2}|^2 + 2\sum _{i=1}^n c|\nabla x_i^{1/2}|^2 \nonumber \\&= 2|\nabla c^{1/2}|^2 + 2c\sum _{i=1}^n |\nabla x_i^{1/2}|^2 \leqslant C\sum _{i=1}^n |\nabla x_i^{1/2}|^2, \end{aligned}$$

where $C>0$ depends only on $M_\mathrm{min}$ and $M_{\mathrm{max}}$. Adding (33) and (34) and integrating over $\Omega$ then shows that, for another constant $C>0$,

$$\begin{aligned} \sum _{i=1}^n\int _\Omega |\nabla x_i^{1/2}|^2 \geqslant C\bigg (\sum _{i=1}^n\int _\Omega |\nabla c_i^{1/2}|^2 \hbox {d}z + \int _\Omega |\nabla c^{1/2}|^2 \hbox {d}z\bigg ). \end{aligned}$$

The lemma then follows from definition (12) of $D[{\varvec{x}}]$. $\quad \square$

#### Lemma 18

There exists a constant $C_\mathrm{CKP}>0$, only depending on $M_{\mathrm{max}}$, such that

$$\begin{aligned} E[{\varvec{x}}|{\varvec{x}}_\infty ] \geqslant C_\mathrm{CKP}\sum _{i=1}^n \Vert x_i-x_{i\infty }\Vert _{L^1(\Omega )}^2. \end{aligned}$$

#### Proof

The estimate is a consequence of the Csiszár–Kullback–Pinsker inequality. Since we are interested in the constant, we provide the (short) proof. We recall that $1/M_{\mathrm{max}}\leqslant c\leqslant 1/M_\mathrm{min}$. Arguing as in (31) and using $\Phi (y)\geqslant 1$ for $y\in {\mathbb {R}}_+$, we obtain

$$\begin{aligned} E[{\varvec{x}}|{\varvec{x}}_\infty ]&= \sum _{i=1}^n\int _\Omega cx_{i\infty }\bigg (\frac{x_i}{x_{i\infty }} \ln \frac{x_i}{x_{i\infty }} - \frac{x_i}{x_{i\infty }} + 1\bigg )\hbox {d}z \\&= \sum _{i=1}^n\int _\Omega cx_{i\infty }\Phi \bigg (\frac{x_i}{x_{i\infty }}\bigg ) \bigg (\bigg (\frac{x_i}{x_{i\infty }}\bigg )^{1/2}-1\bigg )^2 \hbox {d}z \\&\geqslant \frac{1}{M_{\mathrm{max}}}\sum _{i=1}^n\int _\Omega (x_i^{1/2}-x_{i\infty }^{1/2})^2 \hbox {d}z. \end{aligned}$$

Then, by the Cauchy–Schwarz inequality and the bounds $x_i\leqslant 1$, $x_{i\infty }\leqslant 1$,

$$\begin{aligned} E[{\varvec{x}}|{\varvec{x}}_\infty ]&\geqslant \frac{1}{M_{\mathrm{max}}}\sum _{i=1}^n\bigg (\int _\Omega |x_i^{1/2}-x_{i\infty }^{1/2}| \hbox {d}z\bigg )^2 \\&= \frac{1}{M_{\mathrm{max}}}\sum _{i=1}^n\bigg (\int _\Omega \frac{|x_i-x_{i\infty }|}{x_i^{1/2}+x_{i\infty }^{1/2}}\hbox {d}z\bigg )^2 \\&\geqslant \frac{1}{4M_{\mathrm{max}}}\sum _{i=1}^n\bigg (\int _\Omega |x_i-x_{i\infty }|\hbox {d}z\bigg )^2. \end{aligned}$$

This finishes the proof. $\quad \square$

### The Case of Equal Homogeneities

The aim of this and the following subsection is the proof of the functional inequality $D[{\varvec{x}}]\geqslant \lambda E[{\varvec{x}}|{\varvec{x}}_\infty ]$ for some $\lambda >0$. For this, we will distinguish two cases, the case which we call *equal homogeneities*,

35 $$\begin{aligned} \sum _{i=1}^n\alpha _i^a = \sum _{i=1}^n\beta _i^a \quad \text{ for } \text{ all } a=1,\ldots ,N, \end{aligned}$$

and the case of *unequal homogeneities*, for which exists $a \in \{1, \ldots , N\}$ such that

36 $$\begin{aligned} \sum _{i=1}^n\alpha _i^a \ne \sum _{i=1}^n\beta _i^a. \end{aligned}$$

This subsection is concerned with the first case.

#### Proposition 19

(Entropy entropy-production inequality; case of equal homogeneities) Fix ${{\varvec{M}}^0}\in {\mathbb {R}}_+^m$ such that ${\varvec{\zeta }}{{\varvec{M}}^0}= 1$. Let ${\varvec{x}}_\infty$ be the equilibrium constructed in Theorem 11. Assume that (35) holds and system (1)–(2) has no boundary equilibria. Then there exists a constant $\lambda >0$, which is constructive up to a finite-dimensional inequality, such that

$$\begin{aligned} D[{\varvec{x}}]\geqslant \lambda E[{\varvec{x}}|{\varvec{x}}_\infty ] \end{aligned}$$

for all functions ${\varvec{x}}: \Omega \rightarrow {\mathbb {R}}_+^n$ having the same regularity as the corresponding solutions in Theorem 4, and satisfying ${\mathbb {Q}}\overline{{\varvec{c}}} = {{\varvec{M}}^0}$.

#### Proof

We use Lemma 16 and the Poincaré inequality to obtain

$$\begin{aligned} E[{\varvec{x}}|{\varvec{x}}_\infty ]&\leqslant C\sum _{i=1}^n\bigg (\int _\Omega \Big (c_i^{1/2}-\overline{c_i^{1/2}}\,\Big )^2 \hbox {d}z + \big (\overline{c_i}^{1/2}-c_{i\infty }^{1/2}\big )^2\bigg ) \\&\leqslant C\sum _{i=1}^n\bigg \{\int _\Omega |\nabla c_i^{1/2}|^2 \hbox {d}z + \bigg (\bigg (\frac{\overline{c_i}}{c_{i\infty }}\bigg )^{1/2}-1\bigg )^2\bigg \}. \end{aligned}$$

Next, we take into account estimate [25, formula (11)] and [25, Lemma 2.7]:

37 $$\begin{aligned} E[{\varvec{x}}|{\varvec{x}}_\infty ]&\leqslant C\sum _{i=1}^n\int _\Omega |\nabla c_i^{1/2}|^2 \hbox {d}z + \frac{C}{H_1}\sum _{a=1}^N\bigg \{\bigg (\sqrt{\frac{\overline{{\varvec{c}}}}{{\varvec{c}}_\infty }} \bigg )^{{\varvec{\alpha ^a}}} - \bigg (\sqrt{\frac{\overline{{\varvec{c}}}}{{\varvec{c}}_\infty }} \bigg )^{{\varvec{\beta ^a}}}\bigg \}^2 \nonumber \\&\leqslant C\sum _{i=1}^n\int _\Omega |\nabla c_i^{1/2}|^2 \hbox {d}z + C\sum _{a=1}^N\big (k_f^a{\varvec{c}}^{{\varvec{\alpha }}^a}-k_b^a{\varvec{c}}^{{\varvec{\beta }}^a}\big ) \ln \frac{k_f^a{\varvec{c}}^{{\varvec{\alpha }}^a}}{k_b^a{\varvec{c}}^{{\varvec{\beta }}^a}}, \end{aligned}$$

where $H_1>0$ is the constant in the finite-dimensional inequality (11) of [25]. Observe that we can apply the results [25] since ${\mathbb {Q}}\overline{{\varvec{c}}}={{\varvec{M}}^0}$ is satisfied; see Lemma 9.

We claim that the last term is smaller or equal $D[{\varvec{x}}]$. Indeed, inserting the expression $x_i=c_i/c$ in the last term of the entropy production (12) and employing assumption (35), it follows that

38 $$\begin{aligned} \sum _{a=1}^N\int _\Omega (k_f^a{\varvec{x}}^{{\varvec{\alpha }}^a}-k_b^a{\varvec{x}}^{{\varvec{\beta }}^a}) \ln \frac{k_f^a {\varvec{x}}^{{\varvec{\alpha }}^a}}{k_b^a {\varvec{x}}^{{\varvec{\beta }}^a}}\hbox {d}z&= \sum _{a=1}^N\int _\Omega \frac{1}{c^{\alpha _1^a+\cdots \alpha _n^a}} (k_f^a{\varvec{c}}^{{\varvec{\alpha }}^a}-k_b^a{\varvec{c}}^{{\varvec{\beta }}^a}) \ln \frac{k_f^a {\varvec{c}}^{{\varvec{\alpha }}^a}}{k_b^a {\varvec{c}}^{{\varvec{\beta }}^a}}\hbox {d}z \nonumber \\&\geqslant C\sum _{a=1}^N\int _\Omega (k_f^a{\varvec{c}}^{{\varvec{\alpha }}^a}-k_b^a{\varvec{c}}^{{\varvec{\beta }}^a}) \ln \frac{k_f^a {\varvec{c}}^{{\varvec{\alpha }}^a}}{k_b^a {\varvec{c}}^{{\varvec{\beta }}^a}}\hbox {d}z, \end{aligned}$$

where we used in the last step $M_\mathrm{min}\leqslant 1/c\leqslant M_{\mathrm{max}}$. By Lemma 17, this shows that

$$\begin{aligned} D[{\varvec{x}}] \geqslant C\sum _{i=1}^n\int _\Omega |\nabla c_i^{1/2}|^2 \hbox {d}z + C\sum _{a=1}^N\int _\Omega (k_f^a{\varvec{c}}^{{\varvec{\alpha }}^a}-k_b^a{\varvec{c}}^{{\varvec{\beta }}^a}) \ln \frac{k_f^a {\varvec{c}}^{{\varvec{\alpha }}^a}}{k_b^a {\varvec{c}}^{{\varvec{\beta }}^a}}\hbox {d}z, \end{aligned}$$

and combining this estimate with (37) concludes the proof. $\quad \square$

### The Case of Unequal Homogeneities

In this subsection, we consider the case (36) of unequal homogeneities. Since we cannot replace ${\varvec{x}}$ easily by ${\varvec{c}}$ as in (38), the estimates are much more involved than in the case of equal homogeneities. Similar as to Section 3.2, our idea is to introduce *c* as a new variable and to lift the problem from the *n* variables $c_1,\ldots ,c_n$ to the $n+1$ variables $c_1,\ldots ,c_n,c$. Then $D[{\varvec{x}}]$ is represented by $n+1$ variables $c_1,\ldots ,c_n,c$ under the conservation laws ${\mathbb {Q}}\overline{{\varvec{c}}}={{\varvec{M}}^0}$ and the additional constraint $c=\sum _{i=1}^n c_i$ and thus $\overline{c}=\sum _{i=1}^n \overline{c_i}$. We employ the notation (21) and (22).

First, let $\gamma ^a:=\sum _{i=1}^n(\alpha _i^a-\beta _i^a)$ and assume that $\gamma ^a\geqslant 0$. With the definitions $x_i=c_i/c$, $\omega _i=c_i$ for $i=1,\ldots ,n$, and $\omega _{n+1}=c$, we compute

$$\begin{aligned} \sum _{a=1}^N&\int _\Omega (k_f^a{\varvec{x}}^{{\varvec{\alpha }}^a}-k_b^a{\varvec{x}}^{{\varvec{\beta }}^a}) \ln \frac{k_f^a {\varvec{x}}^{{\varvec{\alpha }}^a}}{k_b^a {\varvec{x}}^{{\varvec{\beta }}^a}}\hbox {d}z \\&= \sum _{a=1}^N\int _\Omega \bigg \{k_f^a\prod _{i=1}^n\bigg (\frac{c_i}{c} \bigg )^{\alpha _i^a} - k_b^a\prod _{i=1}^n\bigg (\frac{c_i}{c}\bigg )^{\beta _i^a}\bigg \} \ln \frac{k_f^a\prod _{i=1}^n(c_i/c)^{\alpha _i^a}}{k_b^a\prod _{i=1}^n (c_i/c)^{\beta _i^a}}\hbox {d}z\\&= \sum _{a=1}^N\int _\Omega \frac{1}{c^{\sum _{i=1}^n\alpha _i^a}} \bigg (k_f^a\prod _{i=1}^n c_i^{\alpha _i^a} - k_b^ac^{\gamma ^a}\prod _{i=1}^n c_i^{\beta _i}\bigg ) \ln \frac{k_f^a\prod _{i=1}^n c_i^{\alpha _i^a}}{k_b^ac^{\gamma ^a} \prod _{i=1}^n c_i^{\beta _i}} \hbox {d}z \\&= \sum _{a=1}^N\int _\Omega \frac{1}{c^{\sum _{i=1}^n\alpha _i^a}} \big (k_f^a{\varvec{\omega }}^{{\varvec{\mu }}^a} - k_b^a{\varvec{\omega }}^{{\varvec{\nu }}^a}\big ) \ln \frac{k_f^a{\varvec{\omega }}^{{\varvec{\mu }}^a}}{k_b^a{\varvec{\omega }}^{{\varvec{\nu }}^a}} \hbox {d}z \\&\geqslant C\int _\Omega \big (k_f^a{\varvec{\omega }}^{{\varvec{\mu }}^a} - k_b^a{\varvec{\omega }}^{{\varvec{\nu }}^a}\big ) \ln \frac{k_f^a{\varvec{\omega }}^{{\varvec{\mu }}^a}}{k_b^a{\varvec{\omega }}^{{\varvec{\nu }}^a}} \hbox {d}z, \end{aligned}$$

where $C>0$ depends on $M_{\mathrm{max}}$. In the case $\gamma ^a < 0$, we argue in the same way, leading to

$$\begin{aligned} \sum _{a=1}^N\int _\Omega (k_f^a{\varvec{x}}^{{\varvec{\alpha }}^a}-k_b^a{\varvec{x}}^{{\varvec{\beta }}^a}) \ln \frac{k_f^a {\varvec{x}}^{{\varvec{\alpha }}^a}}{k_b^a {\varvec{x}}^{{\varvec{\beta }}^a}}\hbox {d}z&= \sum _{a=1}^N\int _\Omega \frac{1}{c^{\sum _{i=1}^n\beta _i^a}} \big (k_f^a{\varvec{\omega }}^{{\varvec{\mu }}^a} - k_b^a{\varvec{\omega }}^{{\varvec{\nu }}^a}\big ) \ln \frac{k_f^a{\varvec{\omega }}^{{\varvec{\mu }}^a}}{k_b^a{\varvec{\omega }}^{{\varvec{\nu }}^a}} \hbox {d}z \\&\geqslant C\int _\Omega \big (k_f^a{\varvec{\omega }}^{{\varvec{\mu }}^a} - k_b^a{\varvec{\omega }}^{{\varvec{\nu }}^a}\big ) \ln \frac{k_f^a{\varvec{\omega }}^{{\varvec{\mu }}^a}}{k_b^a{\varvec{\omega }}^{{\varvec{\nu }}^a}} \hbox {d}z. \end{aligned}$$

Consequently, taking into account Lemma 17, we find that

39 $$\begin{aligned} D[{\varvec{x}}] \geqslant \widetilde{D}[{\varvec{\omega }}] := C\sum _{i=1}^{n+1}\int _\Omega |\nabla \omega _i^{1/2}|^2\hbox {d}z + C\sum _{a=1}^N\int _\Omega \big (k_f^a{\varvec{\omega }}^{{\varvec{\mu }}^a} -k_b^a{\varvec{\omega }}^{{\varvec{\nu }}^a}\big ) \ln \frac{k_f^a {\varvec{\omega }}^{{\varvec{\mu }}^a}}{k_b^a {\varvec{\omega }}^{{\varvec{\nu }}^a}}\hbox {d}z. \end{aligned}$$

We need to determine the conservation laws for $\overline{{\varvec{\omega }}}$. We write ${\varvec{1}}=(1,\ldots ,1)^\top \in {\mathbb {R}}^{n+1}$.

#### Lemma 20

Assume that ${\mathbb {Q}}\overline{{\varvec{c}}}={{\varvec{M}}^0}$. Then $\overline{{\varvec{\omega }}}=(\overline{c_1},\ldots ,\overline{c_n},\overline{c})$ satisfies the conservation laws

$$\begin{aligned} \widehat{{\mathbb {Q}}}\overline{{\varvec{\omega }}} = {\widehat{{\varvec{M}}}}^0, \end{aligned}$$

where $\widehat{{\mathbb {Q}}}$ and ${\widehat{{\varvec{M}}}}^0$ are defined by

40 $$\begin{aligned} \widehat{{\mathbb {Q}}} = \begin{pmatrix} {\mathbb {Q}}&{} {\varvec{0}} \\ {\varvec{1}}^\top &{} -1 \end{pmatrix}\in {\mathbb {R}}^{(m+1)\times (n+1)}, \quad {\widehat{{\varvec{M}}}}^0= \begin{pmatrix} {{\varvec{M}}^0}\\ 0 \end{pmatrix}\in {\mathbb {R}}^{n+1}. \end{aligned}$$

#### Proof

The result follows from a direct computation:

$$\begin{aligned} \widehat{{\mathbb {Q}}}\overline{{\varvec{\omega }}} = \begin{pmatrix} {\mathbb {Q}}&{} {\varvec{0}} \\ {\varvec{1}} &{} -1 \end{pmatrix} \begin{pmatrix} \overline{\omega _1} \\ \vdots \\ \overline{\omega _n} \\ \overline{\omega _{n+1}} \end{pmatrix} = \begin{pmatrix} {\mathbb {Q}}\overline{{\varvec{c}}} \\ \sum _{i=1}^n \overline{c_i} - \overline{c} \end{pmatrix} = \begin{pmatrix} {{\varvec{M}}^0}\\ 0 \end{pmatrix}, \end{aligned}$$

since it holds that $\overline{c}=\sum _{i=1}^n\overline{c_i}$. $\quad \square$

#### Lemma 21

There exists a constant $C>0$, depending on $\Omega$, *n*, *N*, $k_f^a$, $k_b^a$ ($a=1,\ldots ,N$), and $M_i$ ($i=1,\ldots ,n$), such that

$$\begin{aligned} \widetilde{D}[{\varvec{\omega }}] \geqslant C\sum _{a=1}^N\Big ( (k_f^a)^{1/2}\sqrt{\overline{{\varvec{\omega }}}}^{{\varvec{\mu }}^a} - (k_b^a)^{1/2}\sqrt{\overline{{\varvec{\omega }}}}^{{\varvec{\nu }}^a}\Big )^2 \end{aligned}$$

for all measurable functions ${\varvec{\omega }}: \Omega \rightarrow {\mathbb {R}}_+^{n+1}$ such that $\widetilde{D}[{\varvec{\omega }}]$ is finite, with $\widetilde{D}[{\varvec{\omega }}]$ defined in (39).

A similar but slightly simpler result for reaction–diffusion systems is proved in [25, Lemma 2.7]. The proof of this lemma is lengthy and therefore shifted to 6. We remark that the validity of this lemma applies to all measurable functions with $\widetilde{D}[{\varvec{\omega }}] < +\infty$.

#### Lemma 22

Assume that (1)–(2) possesses no boundary equilibria. Fix ${{\varvec{M}}^0}\in {\mathbb {R}}_+^m$ such that ${\varvec{\zeta }}{{\varvec{M}}^0}= 1$. Then there exists a nonconstructive constant $C>0$ such that for all $\overline{{\varvec{ \omega }}}\in {\mathbb {R}}_+^{n+1}$ satisfying $\widehat{{\mathbb {Q}}}\overline{{\varvec{\omega }}}={\widehat{{\varvec{M}}}}^0$, it holds that

41 $$\begin{aligned} \sum _{a=1}^N\Big ((k_f^a)^{1/2}\sqrt{\overline{{\varvec{\omega }}}}^{{\varvec{\mu }}^a} - (k_b^a)^{1/2}\sqrt{\overline{{\varvec{\omega }}}}^{{\varvec{\nu }}^a}\Big )^2 \geqslant C\sum _{i=1}^{n+1}\big (\overline{\omega _i}^{1/2}-\omega _{i\infty }^{1/2}\big )^2, \end{aligned}$$

where ${\varvec{\omega }}_\infty$ is constructed in Proposition 12.

#### Remark 23

We mark that this lemma is proved for *any* vector $\overline{{\varvec{\omega }}}\in {\mathbb {R}}_+^{n+1}$ satisfying the conservation laws. It does not use any analytical properties of solutions to (1)–(2). The notation $\overline{{\varvec{\omega }}}$ is a bit abusive, since we later apply this lemma to the average $\overline{{\varvec{\omega }}}$, where ${\varvec{\omega }}$ is constructed from solutions to (1)–(2).

#### Remark 24

While all the constants before and after this lemma are constructive, this is not the case for the constant in Lemma 22, since the lemma is proved by using a contradiction argument. Still, inequality (41) is finite-dimensional. Therefore, in the general case, the rate of convergence to equilibrium to system (1)–(2) is constructive up to the finite-dimensional inequality (41). We present in Section 4 an example for which (41) can be proved with a constructive (even explicit) constant, which consequently leads to a constructive rate of convergence to equilibrium for (1)–(2). $\quad \square$

#### Proof of Lemma 22

We first show that ${\varvec{\overline{\omega }}}$ is bounded. Indeed, we infer from $\widehat{{\mathbb {Q}}}\overline{{\varvec{\omega }}} = \widehat{{\varvec{M}}}^0$ that ${\mathbb {Q}}\overline{{\varvec{\omega }}}' = {{\varvec{M}}^0}$. Thus, $1 = {\varvec{\zeta }}{{\varvec{M}}^0}= {\varvec{\zeta }}{\mathbb {Q}}\overline{{\varvec{\omega }}} = \sum _{i=1}^{n}M_i \overline{\omega _i}$. Hence, $\overline{\omega _i} \leqslant 1/M_\mathrm{min}$ and consequently $\overline{\omega }_{n+1} = \sum _{i=1}^{n}\overline{\omega _i} \leqslant n/M_\mathrm{min}$.

We will now prove that

$$\begin{aligned} \lambda := \inf _{\overline{{\varvec{\omega }}}\in {\mathbb {R}}_+^{n+1}: \widehat{{\mathbb {Q}}}\overline{{\varvec{\omega }}}={\widehat{{\varvec{M}}}}^0} \frac{\sum _{a=1}^N\big ((k_f^a)^{1/2}\sqrt{\overline{{\varvec{\omega }}}}^{{\varvec{\mu }}^a} - (k_b^a)^{1/2}\sqrt{\overline{{\varvec{\omega }}}}^{{\varvec{\nu }}^a}\big )^2}{ \sum _{i=1}^{n+1}\big (\overline{\omega _i}^{1/2}-\omega _{i\infty }^{1/2}\big )^2} > 0. \end{aligned}$$

It is obvious that $\lambda \geqslant 0$. Since the denominator is bounded from above, $\lambda =0$ can occur only if the nominator approaches zero. In view of Proposition 12 and the fact that the system is assumed to have no boundary equilibria, the nominator can converge to zero only when $\overline{{\varvec{\omega }}}\rightarrow {\varvec{\omega }}_\infty$. Therefore, $\lambda =0$ is only possible if $\delta =0$, where $\delta$ is the linearized version of $\lambda$ defined in Lemma 25 below. Setting $\eta _i=\overline{\omega _i}-\omega _{i\infty }$, Lemma 25 shows that $\delta =0$ if and only if

$$\begin{aligned} 0 = \liminf _{\widehat{{\mathbb {Q}}}\overline{{\varvec{\omega }}}={\widehat{{\varvec{M}}}}^0,\, \overline{{\varvec{\omega }}}\rightarrow {\varvec{\omega }}_\infty } \frac{\sum _{a=1}^N k_f^a{\varvec{\omega }}_\infty ^{{\varvec{\mu }}^a}\big \{\sum _{i=1}^{n+1} (\mu _i^a-\nu _i^a)\eta _i\omega _{i\infty }^{-1}\big \}^2}{ \sum _{i=1}^{n+1}\eta _i^2\omega _{i\infty }^{-1}}. \end{aligned}$$

Since the nominator and denominator have the same homogeneity, the limit inferior remains unchanged if ${\varvec{\eta }}=(\eta _1,\ldots ,\eta _{n+1})$ has unit length, $\Vert {\varvec{\eta }}\Vert _{{\mathbb {R}}^{n+1}}=1$ (using the Euclidean norm). We infer from $\widehat{{\mathbb {Q}}}\overline{{\varvec{\omega }}}={\widehat{{\varvec{M}}}}^0=\widehat{{\mathbb {Q}}} {\varvec{\omega }}_\infty$ that $\widehat{{\mathbb {Q}}}{\varvec{\eta }}=0$. Hence, we have $\delta =0$ if and only if there exists a vector ${\varvec{\eta }}\in {\mathbb {R}}^{n+1}$ satisfying $\Vert {\varvec{\eta }}\Vert _{{\mathbb {R}}^{n+1}}=1$, $\widehat{{\mathbb {Q}}}{\varvec{\eta }}=0$, and

$$\begin{aligned} \sum _{i=1}^{n+1}(\mu _i^a-\nu _i^a)\frac{\eta _i}{\omega _{i\infty }} = 0 \quad \text{ for } \text{ all } a=1,\ldots ,N. \end{aligned}$$

The last identity implies that the vector ${\varvec{\eta }}/{\varvec{\omega }}_\infty := (\eta _1/\omega _{1\infty },\ldots ,\eta _{n+1}/\omega _{n+1,\infty })^\top$ belongs to the kernel of $\mathbb {P}^\top$, where

$$\begin{aligned} \mathbb {P} = \big ({\varvec{\nu }}^a-{\varvec{\mu }}^a\big )_{a=1,\ldots ,N} \in {\mathbb {R}}^{(n+1)\times N}. \end{aligned}$$

Since the rows of ${\mathbb {Q}}$ form a basis of the Wegscheider matrix $\mathbb {W}=({\varvec{\beta }}^a-{\varvec{\alpha }}^a)_{a=1,\ldots ,N}$, and taking into account definition (22) of ${\varvec{\mu }}^a$ and ${\varvec{\nu }}^a$, we see that the columns of the matrix

$$\begin{aligned} {\mathbb {Q}}^*:=\begin{pmatrix} {\mathbb {Q}}^\top &{} \mathbf {1}_n \\ \mathbf {0} &{} 1 \end{pmatrix} \end{aligned}$$

form a basis of ${\text {ker}}(\mathbb {P}^\top )$. We deduce that there exists ${\varvec{\rho }}\in {\mathbb {R}}^{n+1}$ such that ${\varvec{\eta }}/{\varvec{\omega }}_\infty ={\mathbb {Q}}^*{\varvec{\rho }}$ or, equivalently, ${\varvec{\eta }} = {\mathbb {D}}{\mathbb {Q}}^*{\varvec{\rho }}$, where ${\mathbb {D}}={\text {diag}}(\omega _{1\infty }, \ldots ,\omega _{n+1,\infty })$. Hence, because of $\widehat{\mathbb {Q}}{\varvec{\eta }}=0$, we obtain $\widehat{\mathbb {Q}}{\mathbb {D}}{\mathbb {Q}}^*{\varvec{\rho }}=0$. The idea is now to prove that ${\varvec{\rho }}=0$, which implies that ${\varvec{\eta }} = {\mathbb {D}}{\mathbb {Q}}^*{\varvec{\rho }}=0$, contradicting $\Vert {\varvec{\eta }}\Vert _{{\mathbb {R}}^{n+1}}=1$.

We claim that the matrix $\widehat{\mathbb {Q}}{\mathbb {D}}{\mathbb {Q}}^*$ is invertible. Indeed, setting $\mathbb {A}_\infty ={\text {diag}}(\omega _{1\infty }, \ldots , \omega _{n\infty })$, we compute

$$\begin{aligned} \widehat{\mathbb {Q}}{\mathbb {D}}{\mathbb {Q}}^* = \begin{pmatrix} {\mathbb {Q}}&{} \mathbf {0} \\ \mathbf {1}^\top &{} -1 \end{pmatrix} \begin{pmatrix} \mathbb {A}_\infty &{} \mathbf {0} \\ \mathbf {0} &{} \omega _{n+1,\infty } \end{pmatrix} \begin{pmatrix} {\mathbb {Q}}^\top &{} \mathbf {1} \\ \mathbf {0} &{} 1 \end{pmatrix} = \begin{pmatrix} {\mathbb {Q}}\mathbb {A}_\infty {\mathbb {Q}}^\top &{} {\mathbb {Q}}\mathbb {A}_\infty \mathbf {1} \\ \mathbf {1}^\top \mathbb {A}_\infty {\mathbb {Q}}^\top &{} \mathbf {1}^\top \mathbb {A}_\infty \mathbf {1} - \omega _{n+1,\infty } \end{pmatrix}. \end{aligned}$$

Since $\mathbf {1}^\top \mathbb {A}_\infty \mathbf {1}=\sum _{i=1}^{n}\omega _{i\infty } =\omega _{n+1,\infty }$ (see Proposition 12), it follows that

$$\begin{aligned} \widehat{\mathbb {Q}}{\mathbb {D}}{\mathbb {Q}}^* = \begin{pmatrix} {\mathbb {Q}}\mathbb {A}_\infty {\mathbb {Q}}^\top &{} {\mathbb {Q}}\mathbb {A}_\infty \mathbf {1} \\ \mathbf {1}^\top \mathbb {A}_\infty {\mathbb {Q}}^\top &{} 0 \end{pmatrix}. \end{aligned}$$

We claim that the matrix ${\mathbb {Q}}\mathbb {A}_\infty {\mathbb {Q}}^\top$ is regular. Since ${\mathbb {Q}}$ has full rank, so is ${\mathbb {Q}}^\top$, and we infer for all ${\varvec{\xi }}\in {\mathbb {R}}^m$ that

$$\begin{aligned} \big \langle {\varvec{\xi }},{\mathbb {Q}}\mathbb {A}_\infty {\mathbb {Q}}^\top {\varvec{\xi }}\big \rangle = \big \langle {\varvec{\xi }}, {\mathbb {Q}}\mathbb {A}_\infty ^{1/2}\mathbb {A}_\infty ^{1/2}{\mathbb {Q}}^\top {\varvec{\xi }}\big \rangle = \big \langle \mathbb {A}_\infty ^{1/2}{\mathbb {Q}}^\top {\varvec{\xi }}, \mathbb {A}_\infty ^{1/2}{\mathbb {Q}}^\top {\varvec{\xi }}\big \rangle \geqslant 0 \end{aligned}$$

with equality if and only if ${\varvec{\xi }}={\varvec{0}}$. Hence, ${\mathbb {Q}}\mathbb {A}_\infty {\mathbb {Q}}^\top$ is regular. Together with the rule on the determinant of block matrices, this shows that

$$\begin{aligned} \det (\widehat{\mathbb {Q}}{\mathbb {D}}{\mathbb {Q}}^*) = \det ({\mathbb {Q}}\mathbb {A}_\infty {\mathbb {Q}}^\top )\det \big [0-(\mathbf {1}^\top \mathbb {A}_\infty {\mathbb {Q}}^\top ) ({\mathbb {Q}}\mathbb {A}_\infty {\mathbb {Q}}^\top )^{-1}({\mathbb {Q}}\mathbb {A}_\infty \mathbf {1})\big ]. \end{aligned}$$

As we already know that $\det ({\mathbb {Q}}\mathbb {A}_\infty {\mathbb {Q}}^\top )\ne 0$, it remains to verify that the second factor does not vanish. As the expression in the brackets $[\cdots ]$ is a number, we need to show that

42 $$\begin{aligned} (\mathbf {1}^\top \mathbb {A}_\infty {\mathbb {Q}}^\top ) ({\mathbb {Q}}\mathbb {A}_\infty {\mathbb {Q}}^\top )^{-1}({\mathbb {Q}}\mathbb {A}_\infty \mathbf {1}) \ne 0. \end{aligned}$$

The diagonal matrix $\mathbb {A}_\infty \in {\mathbb {R}}^{n\times n}$ has strictly positive diagonal elements. Therefore, (42) is equivalent to

$$\begin{aligned} (\mathbf {1}^\top \mathbb {A}_\infty ^{1/2})(\mathbb {A}_\infty ^{1/2}{\mathbb {Q}}^\top ) \big (({\mathbb {Q}}\mathbb {A}_\infty ^{1/2})(\mathbb {A}_\infty ^{1/2}{\mathbb {Q}}^\top )\big )^{-1} ({\mathbb {Q}}\mathbb {A}_\infty ^{1/2})(\mathbf {1}^\top \mathbb {A}_\infty ^{1/2})^\top \ne 0. \end{aligned}$$

We abbreviate the left-hand side by introducing ${\varvec{z}}=\mathbf {1}^\top \mathbb {A}_\infty ^{1/2}\in {\mathbb {R}}^{1\times n}$ and $\mathbb {X}=\mathbb {A}_\infty ^{1/2}{\mathbb {Q}}^\top \in {\mathbb {R}}^{n\times m}$. Then (42) becomes

$$\begin{aligned} {\varvec{z}}\mathbb {X}(\mathbb {X}^\top \mathbb {X})^{-1}\mathbb {X}^\top {\varvec{z}}^\top \ne 0. \end{aligned}$$

Since $\mathbb {X}$ is not a square matrix, we cannot invert it, but we may consider its Moore-Penrose generalized inverse $\mathbb {X}^\dag$; see [45] or [49, Section 11.5] for a definition and properties. We compute

$$\begin{aligned} {\varvec{z}}\mathbb {X}(\mathbb {X}^\top \mathbb {X})^{-1}\mathbb {X}^\top {\varvec{z}}^\top&= {\varvec{z}}\mathbb {X}(\mathbb {X}^\top \mathbb {X})^\dag \mathbb {X}^\top {\varvec{z}}^\top \qquad \text{[49, } \text{ page } \text{218] } \\&= {\varvec{z}}\mathbb {X}\mathbb {X}^\dag (\mathbb {X}^\top )^\dag \mathbb {X}^\top {\varvec{z}}^\top \qquad \text{[45, } \text{ Lemma } \text{1.5] } \\&= {\varvec{z}}\mathbb {X}\mathbb {X}^\dag (\mathbb {X}^\dag )^\top \mathbb {X}^\top {\varvec{z}}^\top \qquad \text{[49, } \text{ Prop. } \text{11.5] } \\&= {\varvec{z}}(\mathbb {X}\mathbb {X}^\dag )(\mathbb {X}\mathbb {X}^\dag )^\top {\varvec{z}}^\top \qquad \text{[45, } \text{ Lemma } \text{1.5] } \\&= \Vert (\mathbb {X}\mathbb {X}^\dag )^\top {\varvec{z}}^\top \Vert _{{\mathbb {R}}^n}^2. \end{aligned}$$

Consequently, (42) holds if and only if $(\mathbb {X}\mathbb {X}^\dag )^\top {\varvec{z}}^\top \ne 0$ or ${\varvec{z}}^\top \not \in {\text {ker}}((\mathbb {X}\mathbb {X}^\dag )^\top )$. Now, it holds that

$$\begin{aligned} {\text {ker}}\big ((\mathbb {X}\mathbb {X}^\dag )^\top \big ) = {\text {ker}}\big ((\mathbb {X}^\dag )^\top \mathbb {X}^\top \big ) = {\text {ker}}\big ((\mathbb {X}^\top )^\dag \mathbb {X}^\top \big ) = {\text {ker}}(\mathbb {X}^\top ), \end{aligned}$$

where the last step follows from [49, page 219]. We infer that ${\varvec{z}}^\top \not \in {\text {ker}}((\mathbb {X}\mathbb {X}^\dag )^\top )$ if and only if $\mathbb {A}_\infty ^{1/2}\mathbf {1}={\varvec{z}}^\top \not \in {\text {ker}}(\mathbb {X}^\top ) ={\text {ker}}({\mathbb {Q}}\mathbb {A}_\infty ^{1/2})$, which is equivalent to

$$\begin{aligned} 0 \ne ({\mathbb {Q}}\mathbb {A}_\infty ^{1/2})(\mathbb {A}_\infty ^{1/2}\mathbf {1}) = {\mathbb {Q}}\mathbb {A}_\infty \mathbf {1} = {\mathbb {Q}}{\varvec{\omega }}_\infty ', \end{aligned}$$

and this property holds true since ${\mathbb {Q}}{\varvec{\omega }}_\infty '={{\varvec{M}}^0}\ne 0$. This proves that (42) holds. As mentioned before, this implies that ${\varvec{\rho }}=0$ and consequently ${\varvec{\eta }}=0$, which contradicts the fact that ${\varvec{\eta }}$ has unit length. We conclude that $\delta >0$ (defined in Lemma 25) and $\lambda >0$, finishing the proof. $\quad \square$

We now provide the technical computations needed in Lemma 22.

#### Lemma 25

Let ${\varvec{\omega }}_\infty$ be a positive detailed-balance equilibrium constructed in Proposition 12. It holds that

$$\begin{aligned} \delta&:= \liminf _{\widehat{{\mathbb {Q}}}\overline{{\varvec{\omega }}}={\widehat{{\varvec{M}}}}^0,\, \overline{{\varvec{\omega }}}\rightarrow {\varvec{\omega }}_\infty } \frac{\sum _{a=1}^N\big \{(k_f^a)^{1/2}\sqrt{\overline{{\varvec{\omega }}}}^{{\varvec{\mu }}^a} - (k_b^a)^{1/2}\sqrt{\overline{{\varvec{\omega }}}}^{{\varvec{\nu }}^a}\big \}^2}{ \sum _{i=1}^{n+1}\big (\overline{\omega _i}^{1/2}-\omega _{i\infty }^{1/2}\big )^2} \\&= \frac{1}{2}\liminf _{\widehat{{\mathbb {Q}}}\overline{{\varvec{\omega }}}={\widehat{{\varvec{M}}}}^0,\, \overline{{\varvec{\omega }}}\rightarrow {\varvec{\omega }}_\infty } \frac{\sum _{a=1}^N k_f^a{\varvec{\omega }}_\infty ^{{\varvec{\mu }}^a}\big \{\sum _{i=1}^{n+1} (\mu _i^a-\nu _i^a)(\overline{\omega _i}-\omega _{i\infty })\omega _{i\infty }^{-1}\big \}^2}{ \sum _{i=1}^{n+1}(\overline{\omega _i}-\omega _{i\infty })^2\omega _{i\infty }^{-1}}. \end{aligned}$$

#### Proof

We denote by

$$\begin{aligned} D_1(\overline{{\varvec{\omega }}})&= \sum _{a=1}^N\Big ((k_f^a)^{1/2}\sqrt{\overline{{\varvec{\omega }}}}^{{\varvec{\mu }}^a} - (k_b^a)^{1/2}\sqrt{\overline{{\varvec{\omega }}}}^{{\varvec{\nu }}^a}\Big )^2, \\ D_2(\overline{{\varvec{\omega }}})&= \sum _{i=1}^{n+1}\big (\overline{\omega _i}^{1/2}-\omega _{i\infty }^{1/2} \big )^2 \end{aligned}$$

the nominator and denominator of the definition of $\delta$, respectively. We linearize both expressions around ${\varvec{\omega }}_\infty$ as follows:

43 $$\begin{aligned} \begin{aligned} D_i(\overline{{\varvec{\omega }}})&= D_i({\varvec{\omega }}_\infty ) + \nabla D_i({\varvec{\omega }}_\infty ) \cdot (\overline{{\varvec{\omega }}}-{\varvec{\omega }}_\infty ) \\&\phantom {xx}{}+ \frac{1}{2}(\overline{{\varvec{\omega }}}-{\varvec{\omega }}_\infty )^\top \nabla ^2D_i({\varvec{\omega }}_\infty )(\overline{{\varvec{\omega }}}-{\varvec{\omega }}_\infty ) + o(|\overline{{\varvec{\omega }}}-{\varvec{\omega }}_\infty |^2). \end{aligned} \end{aligned}$$

Since ${\varvec{\omega }}_\infty$ is a detailed-balance equilibrium, it holds that $(k_f^a)^{1/2}\sqrt{{\varvec{\omega }}_\infty }^{{\varvec{\mu }}^a} = (k_b^a)^{1/2}\sqrt{{\varvec{\omega }}_\infty }^{{\varvec{\nu }}^a}$ for all $a=1,\ldots ,N$, implying that $D_1({\varvec{\omega }}_\infty )=0$ and $\nabla D_1({\varvec{\omega }}_\infty )=0$. Let $\partial _i=\partial /\partial \omega _i$. Then

$$\begin{aligned}&\partial _j\partial _i D_1(\overline{{\varvec{\omega }}}) \\&\quad = \sum _{a=1}^N\bigg \{\partial _j\partial _i \Big ((k_f^a)^{1/2}\sqrt{\overline{{\varvec{\omega }}}}^{{\varvec{\mu }}^a} - (k_b^a)^{1/2}\sqrt{\overline{{\varvec{\omega }}}}^{{\varvec{\nu }}^a}\Big ) \Big ((k_f^a)^{1/2}\sqrt{\overline{{\varvec{\omega }}}}^{{\varvec{\mu }}^a} - (k_b^a)^{1/2}\sqrt{\overline{{\varvec{\omega }}}}^{{\varvec{\nu }}^a}\Big ) \\&\quad \phantom {xx}{}+ \partial _i \Big ((k_f^a)^{1/2}\sqrt{\overline{{\varvec{\omega }}}}^{{\varvec{\mu }}^a} - (k_b^a)^{1/2}\sqrt{\overline{{\varvec{\omega }}}}^{{\varvec{\nu }}^a}\Big ) \partial _j\Big ((k_f^a)^{1/2}\sqrt{\overline{{\varvec{\omega }}}}^{{\varvec{\mu }}^a} - (k_b^a)^{1/2}\sqrt{\overline{{\varvec{\omega }}}}^{{\varvec{\nu }}^a}\Big )\bigg \}. \end{aligned}$$

The first term vanishes for $\overline{{\varvec{\omega }}}={\varvec{\omega }}_\infty$, and for the second term we compute

$$\begin{aligned}&\partial _i \Big ( (k_f^a)^{1/2}\sqrt{\overline{{\varvec{\omega }}}}^{{\varvec{\mu }}^a} - (k_b^a)^{1/2}\sqrt{\overline{{\varvec{\omega }}}}^{{\varvec{\nu }}^a}\Big ) \\&\quad = (k_f^a)^{1/2}\partial _i\prod _{k=1}^{n+1}\overline{\omega _k}^{\mu _k^a/2} - (k_b^a)^{1/2}\partial _i\prod _{k=1}^{n+1}\overline{\omega _k}^{\nu _k^a/2} \\&\quad = (k_f^a)^{1/2}\frac{\mu _i^a}{2}\frac{1}{\overline{\omega _i}} \prod _{k=1}^{n+1}\overline{\omega _k}^{\mu _k^a/2} - (k_b^a)^{1/2}\frac{\nu _i^a}{2}\frac{1}{\overline{\omega _i}} \prod _{k=1}^{n+1}\overline{\omega _k}^{\nu _k^a/2} \\&\quad = \frac{1}{2\overline{\omega _i}}\Big ((k_f^a)^{1/2}\mu _i^a \sqrt{\overline{{\varvec{\omega }}}}^{{\varvec{\mu }}^a} - (k_b^a)^{1/2}\nu _i^a\sqrt{\overline{{\varvec{\omega }}}}^{{\varvec{\nu }}^a}\Big ). \end{aligned}$$

Evaluating this expression at $\overline{{\varvec{\omega }}}={\varvec{\omega }}_\infty$ and using $(k_f^a)^{1/2}\sqrt{{\varvec{\omega }}_\infty }^{{\varvec{\mu }}^a}=(k_b^a)^{1/2} \sqrt{{\varvec{\omega }}_\infty }^{{\varvec{\nu }}^a}$, it follows that

$$\begin{aligned} \partial _i \Big ((k_f^a)^{1/2}\sqrt{\overline{{\varvec{\omega }}}}^{{\varvec{\mu }}^a} - (k_b^a)^{1/2}\sqrt{\overline{{\varvec{\omega }}}}^{{\varvec{\nu }}^a}\Big ) \Big |_{\overline{{\varvec{\omega }}}={\varvec{\omega }}_\infty } = \frac{1}{2}\frac{\mu _i^a-\nu _i^a}{\omega _{i\infty }} (k_f^a)^{1/2}\sqrt{{\varvec{\omega }}_\infty }^{{\varvec{\mu }}^a}. \end{aligned}$$

Consequently,

$$\begin{aligned} \partial _j\partial _i D_1({\varvec{\omega }}_\infty ) = \frac{1}{4}\sum _{a=1}^N k_f^a{\varvec{\omega }}_\infty ^{{\varvec{\mu }}^a} \frac{\mu _i^a-\nu _i^a}{\omega _{i\infty }} \frac{\mu _j^a-\nu _j^a}{\omega _{j\infty }}, \end{aligned}$$

and the quadratic term in the Taylor expansion becomes at the point ${\varvec{\omega }}_\infty$

$$\begin{aligned} \frac{1}{2}(\overline{{\varvec{\omega }}}-{\varvec{\omega }}_\infty )^\top \nabla ^2D_i({\varvec{\omega }}_\infty )(\overline{{\varvec{\omega }}}-{\varvec{\omega }}_\infty ) = \frac{1}{8}\sum _{a=1}^N k_f^a{\varvec{\omega }}_\infty ^{{\varvec{\mu }}^a}\bigg (\sum _{i=1}^{n+1} \frac{\mu _i^a-\nu _i^a}{\omega _{i\infty }}\big (\overline{\omega _i}-\omega _{i\infty }\big ) \bigg )^2. \end{aligned}$$

Similarly, $$D_2({\varvec{\omega }}_\infty )=0$$, $$\nabla D_2({\varvec{\omega }}_\infty )=0$$, and

$$\begin{aligned} \frac{1}{2}(\overline{{\varvec{\omega }}}-{\varvec{\omega }}_\infty )^\top \nabla ^2D_2({\varvec{\omega }}_\infty )(\overline{{\varvec{\omega }}}-{\varvec{\omega }}_\infty ) = \frac{1}{4}\sum _{i=1}^{n+1}\frac{(\overline{\omega _i}-\omega _{i\infty })^2}{ \omega _{i\infty }}. \end{aligned}$$

We insert these expressions into (43) and compute $D_1(\overline{{\varvec{\omega }}})/D_2(\overline{{\varvec{\omega }}})$. The limit $\overline{{\varvec{\omega }}}\rightarrow {\varvec{\omega }}_\infty$ such that $\widehat{{\mathbb {Q}}}\overline{{\varvec{\omega }}}={\widehat{{\varvec{M}}}}^0$ then gives the conclusion. $\quad \square$

We are ready to prove the main result of this subsection.

#### Proposition 26

(Entropy entropy-production inequality; unequal homogeneities)

Fix ${{\varvec{M}}^0}\in {\mathbb {R}}_+^m$ such that ${\varvec{\zeta }}{{\varvec{M}}^0}= 1$. Let ${\varvec{x}}_\infty$ be the equilibrium constructed in Theorem 11. Assume that (36) holds and system (1)–(2) has no boundary equilibria. Then there exists a constant $\lambda >0$, which is constructive up to a finite-dimensional inequality (in the sense of Remark 24), such that

$$\begin{aligned} D[{\varvec{x}}]\geqslant \lambda E[{\varvec{x}}|{\varvec{x}}_\infty ] \end{aligned}$$

for all functions ${\varvec{x}}: \Omega \rightarrow {\mathbb {R}}_+^n$ having the same regularity as the corresponding solutions in Theorem 4 and satisfying ${\mathbb {Q}}\overline{{\varvec{c}}} = {{\varvec{M}}^0}$.

#### Proof

Lemma 16 shows that

44 $$\begin{aligned} E[{\varvec{x}}|{\varvec{x}}_\infty ] \leqslant C\sum _{i=1}^n\bigg (\int _\Omega \Big (c_i^{1/2}-\overline{c_i^{1/2}}\,\Big )^2 \hbox {d}z + \big (\overline{c_i}^{1/2}-c_{i\infty }^{1/2}\big )^2\bigg ). \end{aligned}$$

The first sum is controlled by $D[{\varvec{x}}]$ using Lemma 17 and the Poincaré inequality (with constant $C_P>0$):

$$\begin{aligned} D[{\varvec{x}}] \geqslant \sum _{i=1}^n\int _\Omega |\nabla c_i^{1/2}|^2 \hbox {d}z \geqslant C_p\sum _{i=1}^n\int _\Omega \big (c_i^{1/2}-\overline{c_i^{1/2}}\big )^2 \hbox {d}z. \end{aligned}$$

The second sum on the right-hand side is estimated by combining estimate (39), Lemmas 21, and 22:

$$\begin{aligned} D[{\varvec{x}}] \geqslant C\sum _{i=1}^{n+1}\big (\overline{\omega _i}^{1/2} -\omega _{i\infty }^{1/2}\big )^2 \geqslant C\sum _{i=1}^n \big (\overline{c_i}^{1/2}-c_{i\infty }^{1/2}\big )^2. \end{aligned}$$

Adding the previous two inequalities and using (44) then concludes the proof. $\quad \square$

### Proof of Theorem 1

The starting point is the discrete entropy inequality (see Remark 8):

$$\begin{aligned} E[{\varvec{x}}^k|{\varvec{x}}_\infty ] + \tau D[{\varvec{x}}^k] + C\varepsilon \tau \sum _{i=1}^{n-1}\Vert w_i^k\Vert _{H^l(\Omega )}^2 \leqslant E[{\varvec{x}}^{k-1}|{\varvec{x}}_\infty ]. \end{aligned}$$

Using the entropy-production inequality from Propositions 19 or 26, this becomes

$$\begin{aligned} E[{\varvec{x}}^k|{\varvec{x}}_\infty ] \leqslant (1+\lambda \tau )^{-1}E[{\varvec{x}}^{k-1}|{\varvec{x}}_\infty ] \end{aligned}$$

and, by induction,

$$\begin{aligned} E[{\varvec{x}}^k|{\varvec{x}}_\infty ] \leqslant (1+\lambda \tau )^{-k}E[{\varvec{x}}^0|{\varvec{x}}_\infty ] = (1+\lambda \tau )^{-T/\tau }E[{\varvec{x}}^0|{\varvec{x}}_\infty ]. \end{aligned}$$

Performing the limit $\tau \rightarrow 0$ or, equivalently, $k\rightarrow \infty$, we find that

$$\begin{aligned} E[{\varvec{x}}(T)|{\varvec{x}}_\infty ]\leqslant \liminf _{k\rightarrow \infty }E[{\varvec{x}}^k|{\varvec{x}}_\infty ] \leqslant e^{-\lambda T}E[{\varvec{x}}^0|{\varvec{x}}_\infty ]. \end{aligned}$$

Clearly, this inequality also holds for $t\in (0,T)$ instead of *T*. Then, by the Csiszár–Kullback–Pinsker inequality in Lemma 18, with constant $C_\mathrm{CKP}>0$,

$$\begin{aligned} \sum _{i=1}^n\Vert x_i(t)-x_{i\infty }\Vert _{L^1(\Omega )}^2 \leqslant \frac{e^{-\lambda t}}{C_\mathrm{CKP}}\int _\Omega h({\varvec{\rho }}'(0))\hbox {d}z. \end{aligned}$$

As $x_i$ is bounded in $L^\infty (0,\infty ;L^\infty (\Omega ))$, we derive the convergence in $L^p$ for $1\leqslant p<\infty$ from an interpolation argument

$$\begin{aligned} \sum _{i=1}^n\Vert x_i(t)-x_{i\infty }\Vert _{L^p(\Omega )}&\leqslant \sum _{i=1}^n\Vert x_i(t)-x_{i\infty }\Vert _{L^\infty (\Omega )}^{1-1/p} \Vert x_i(t)-x_{i\infty }\Vert _{L^1(\Omega )}^{1/p} \\&\leqslant Ce^{-\lambda t/(2p)}, \quad t>0, \end{aligned}$$

which concludes the proof.

## Example: A Specific Reaction

As mentioned in Remark 24, the rate of convergence to equilibrium is generally not constructive since the finite-dimensional inequality (41) is proved by a nonconstructive contradiction argument. The derivation of a constructive constant for this inequality seems to be a challenging problem, which goes beyond the scope of this paper. In this section, we show that, potentially in any specific system, the finite-dimensional inequality (41) can be proved in a constructive way and thus gives the exponential decay with constructive constant. More specifically, we consider the single reversible reaction

$$\begin{aligned} A_1 + A_2 \leftrightharpoons A_3. \end{aligned}$$

We assume for simplicity that the forward and backward reaction constants equal one. Furthermore, $|\Omega |=1$. The corresponding system reads as

45 $$\begin{aligned} \partial _t \rho _1 + {\text {div}}{\varvec{j}}_1&= r_1({\varvec{x}}) = -M_1(x_1x_2 - x_3), \nonumber \\ \partial _t \rho _2 + {\text {div}}{\varvec{j}}_2&= r_2({\varvec{x}}) = -M_2(x_1x_2 - x_3), \nonumber \\ \partial _t \rho _3 + {\text {div}}{\varvec{j}}_3&= r_3({\varvec{x}}) = +M_3(x_1x_2 - x_3). \end{aligned}$$

We conclude from total mass conservation $r_1+r_2+r_3=0$, that $M_1+M_2=M_3$. There are two (formal) conservation laws. The first one follows from

$$\begin{aligned} \frac{\hbox {d}}{\hbox {d}t}\int _\Omega \big (c_1(t)+c_3(t)\big )\hbox {d}z = \frac{\hbox {d}}{\hbox {d}t}\int _\Omega \bigg (\frac{\rho _1(t)}{M_1}+\frac{\rho _3(t)}{M_3}\bigg )\hbox {d}z = 0, \end{aligned}$$

leading to

$$\begin{aligned} \overline{c_1}(t) + \overline{c_3}(t) = M_{13} := \overline{c_1^0} + \overline{c_3^0}, \end{aligned}$$

where $\overline{c_i^0}=\overline{\rho _i^0}/M_i = \int _\Omega \rho _i^0\hbox {d}z/M_i$. The second conservation law reads as

$$\begin{aligned} \overline{c_2}(t) + \overline{c_3}(t) = M_{23} := \overline{c_2^0} + \overline{c_3^0}. \end{aligned}$$

The matrix ${\mathbb {Q}}$ in this case is

$$\begin{aligned} {\mathbb {Q}}= \begin{pmatrix} 1&{} 0 &{} 1 \\ 0 &{} 1 &{} 1 \end{pmatrix}, \end{aligned}$$

and we can choose ${\varvec{\zeta }}=(M_1,M_2)$ since the conservation of total mass, $M_1+M_2=M_3$, gives ${\varvec{\zeta }}{\mathbb {Q}}=(M_1,M_2,M_3)={\varvec{M}}^\top$. The initial mass vector ${{\varvec{M}}^0}=(M_{13},M_{23})^\top$ satisfies ${\varvec{\zeta }}{{\varvec{M}}^0}=M_1M_{13}+M_2M_{23}=1$. It is not difficult to check that the system is detailed balanced and possesses no boundary equilibria, and thus, for any fixed masses $M_{13}>0$, $M_{23}>0$, there exists a unique positive detailed-balance equilibrium ${\varvec{x}}_\infty =(x_{1\infty },x_{2\infty },x_{3\infty })^\top \in (0,1)^3$ satisfying

46 $$\begin{aligned} \begin{aligned}&x_{1\infty }x_{2\infty } = x_{3\infty }, \quad x_{1\infty } + x_{2\infty } + x_{3\infty } = 1, \\&c_{1\infty }+c_{3\infty } = M_{13}, \quad c_{2\infty }+c_{3\infty } = M_{23}, \end{aligned} \end{aligned}$$

where $c_{i\infty }=c_\infty x_{i\infty }$ and $c_\infty =(M_1x_{1\infty } + M_2x_{2\infty } + M_3x_{3\infty })^{-1}$. We claim that we can prove Lemma 22 with a constructive constant. More precisely, we show the following result:

### Lemma 27

There exists a constructive constant $C_0>0$, only depending on $c_{i\infty }$ and the upper bounds of $\overline{c_i}$ ($i=1,2,3$), such that

47 $$\begin{aligned} \big (\sqrt{\overline{c_1}}\sqrt{\overline{c_2}} - \sqrt{\overline{c_3}}\sqrt{\overline{c}}\big )^2 \geqslant C_0\sum _{i=1}^3\big (\sqrt{\overline{c_i}}-\sqrt{c_{i\infty }}\big )^2 \end{aligned}$$

for all nonnegative numbers $\overline{c_i}$ and $\overline{c}$ satisfying

48 $$\begin{aligned}&\overline{c_1} + \overline{c_3} = M_{13} = c_{1\infty }+c_{3\infty }, \nonumber \\&\overline{c_2} + \overline{c_3} = M_{23} = c_{2\infty }+c_{3\infty }, \nonumber \\&\overline{c_1} + \overline{c_2} + \overline{c_3} = \overline{c}. \end{aligned}$$

### Proof

We introduce new variables $\mu _1,\mu _2,\mu _3,\eta \in [-1,\infty )$ by

$$\begin{aligned} \overline{c_i} = c_{i\infty }(1+\mu _i)^2 \quad \text{ for } i=1,2,3, \quad \overline{c} = c_\infty (1+\eta )^2, \end{aligned}$$

recalling that $c_\infty =c_{1\infty }+c_{2\infty }+c_{3\infty }$. The uniform bounds for $\overline{c_i}$ show that there exists a constant $\mu _{\mathrm{max}}>0$ such that $|\mu _i|\leqslant \mu _{\mathrm{max}}$ for $i=1,2,3$. Then the left-hand side of (47) can be formulated as

$$\begin{aligned} \big (\sqrt{\overline{c_1}}\sqrt{\overline{c_2}} - \sqrt{\overline{c_3}}\sqrt{\overline{c}}\big )^2&= \Big (c_{1\infty }^{1/2}c_{2\infty }^{1/2}(1+\mu _1)(1+\mu _2) - c_{3\infty }^{1/2}c_\infty ^{1/2}(1+\mu _3)(1+\eta )\big )^2 \\&= c_{1,\infty }c_{2\infty }\big ((1+\mu _1)(1+\mu _2)-(1+\mu _3)(1+\eta )\big )^2, \end{aligned}$$

where we have used $c_{1\infty }c_{2\infty }=x_{1\infty }x_{2\infty }c_\infty ^2 = x_{3\infty }c_\infty ^2 = c_{3\infty }c_\infty$, which follows from $x_{i\infty }=c_{i\infty }/c_\infty$ and the first equation in (46). Furthermore, the right-hand side of (47) is estimated from above by

$$\begin{aligned} \sum _{i=1}^3\big (\sqrt{\overline{c_i}}-\sqrt{c_{i\infty }}\big )^2 = \sum _{i=1}^3 c_{i\infty }\mu _i^2 \leqslant \max _{i=1,2,3}c_{i\infty }\sum _{i=1}^3 \mu _i^2. \end{aligned}$$

Therefore, it remains to prove the inequality

49 $$\begin{aligned} \big ((1+\mu _1)(1+\mu _2)-(1+\mu _3)(1+\eta )\big )^2 \geqslant C^*\sum _{i=1}^3 \mu _i^2 \end{aligned}$$

for some constructive constant $C^*>0$.

In terms of the new variables $\mu _i$, the conservation laws in (48) can be written as

50 $$\begin{aligned} \begin{aligned} c_{1\infty }(\mu _1^2+2\mu _1) + c_{3\infty }(\mu _3^2+2\mu _3)&= 0, \\ c_{2\infty }(\mu _2^2+2\mu _2) + c_{3\infty }(\mu _3^2+2\mu _3)&= 0. \end{aligned} \end{aligned}$$

Together with the last equation in (48), we obtain

51 $$\begin{aligned} c_{1\infty }(\mu _1^2+2\mu _1) = c_{2\infty }(\mu _2^2+2\mu _2) = c_\infty (\eta ^2+2\eta ). \end{aligned}$$

Since $\mu _i\geqslant -1$ and $\eta \geqslant -1$, we deduce from (50) and (51) that $\mu _1$, $\mu _2$, and $\eta$ always have the same sign and $\mu _3$ has the opposite sign. We consider therefore two cases.

*Case 1:*$\mu _1$, $\mu _2$, $\eta \geqslant 0$*and*$\mu _3\leqslant 0$. Since $\eta ^2+2\eta \geqslant 0$ and $c_\infty =c_{1\infty }+c_{2\infty }+c_{3\infty }$, it follows from (51) that

$$\begin{aligned} c_{1\infty }(\mu _1^2+2\mu _1) = c_\infty (\eta ^2+2\eta ) \geqslant c_{1\infty }(\eta ^2+2\eta ) \end{aligned}$$

and hence $\mu _1\geqslant \eta$ (as $z\mapsto z^2+2z$ is increasing on $[-1,\infty )$). Similarly, we find that $\mu _2\geqslant \eta$. Therefore,

$$\begin{aligned} (1+\mu _1)(1+\mu _2)-(1+\mu _3)(1+\eta ) = (\mu _1-\eta ) + \mu _2 + \mu _1\mu _2 + (-\mu _3) + (-\mu _3)\eta \geqslant 0. \end{aligned}$$

Taking the square of this equation, it follows that

$$\begin{aligned} \big ((1+\mu _1)(1+\mu _2)-(1+\mu _3)(1+\eta )\big )^2&\geqslant \big ((\mu _1-\eta )+\mu _2+(-\mu _3)\big )^2 \\&\geqslant (\mu _1-\eta )^2 + \mu _2^2 + (-\mu _3)^2 \geqslant \mu _2^2+\mu _3^2. \end{aligned}$$

Exchanging the roles of $\mu _1$ and $\mu _2$, we find that

$$\begin{aligned} \big ((1+\mu _1)(1+\mu _2)-(1+\mu _3)(1+\eta )\big )^2 \geqslant \mu _1^2+\mu _3^2. \end{aligned}$$

Adding these inequalities, we have proved (49) with $C^*=\frac{1}{2}$.

*Case 2:*$\mu _1$, $\mu _2$, $\eta \leqslant 0$*and*$\mu _3\geqslant 0$. Because of $\eta ^2+2\eta \leqslant 0$, we have

$$\begin{aligned} c_{1\infty }(\mu _1^2+2\mu _1) = c_\infty (\eta ^2+2\eta ) \leqslant c_{1\infty }(\eta ^2+2\eta ), \end{aligned}$$

which yields $\mu _1\leqslant \eta$. Similarly, $\mu _2\leqslant \eta$. A similar argument as in case 1 leads to

$$\begin{aligned} (1+\mu _3)(1+\eta ) - (1+\mu _1)(1+\mu _2) = \mu _3(1+\eta ) + (\eta -\mu _1) + (-\mu _2)(1+\mu _1) \geqslant 0. \end{aligned}$$

Hence, taking the square,

52 $$\begin{aligned} \big ((1+\mu _1)(1+\mu _2)-(1+\mu _3)(1+\eta )\big )^2&\geqslant \big (\mu _3(1+\eta ) + (\eta -\mu _1) + (-\mu _2)(1+\mu _1)\big )^2 \nonumber \\&\geqslant \mu _3^2(1+\eta )^2. \end{aligned}$$

We deduce from (51) that

$$\begin{aligned} c_\infty (1+\eta )^2 = c_\infty + c_\infty (\eta ^2+2\eta )&= c_\infty + c_{1\infty }(\mu _1^2+2\mu _1) \\&= c_{2\infty } + c_{3\infty } + c_{1\infty }(1+\mu _1)^2. \end{aligned}$$

Consequently, $(1+\eta )^2\geqslant (c_{2\infty }+c_{3\infty })/c_\infty$ and (52) becomes

53 $$\begin{aligned} \big ((1+\mu _1)(1+\mu _2)-(1+\mu _3)(1+\eta )\big )^2 \geqslant \frac{c_{2\infty }+c_{3\infty }}{c_\infty }\mu _3^2. \end{aligned}$$

We infer from $c_{3\infty }(\mu _3^2+2\mu _3)=-c_{1\infty }(\mu _1^2+2\mu _1)$ (see (50)) that

$$\begin{aligned} \mu _3 = \frac{c_{1\infty }(\mu _1+2)}{c_{3\infty }(\mu _3+2)}(-\mu _1) \geqslant \frac{c_{1\infty }}{c_{3\infty }(\mu _{\mathrm{max}}+2)}(-\mu _1) \geqslant 0, \end{aligned}$$

where $\mu _{\mathrm{max}}=\max _{i=1,2,3}\mu _i$. Taking the square gives

$$\begin{aligned} \mu _3^2 \geqslant \frac{c_{1\infty }^2}{c_{3\infty }^2(\mu _{\mathrm{max}}+2)^2}\mu _1^2, \end{aligned}$$

and similarly,

$$\begin{aligned} \mu _3^2 \geqslant \frac{c_{2\infty }^2}{c_{3\infty }^2(\mu _{\mathrm{max}}+2)^2}\mu _2^2. \end{aligned}$$

We employ these bounds in (53) to obtain

$$\begin{aligned} \big ((1+\mu _1)(1+\mu _2)-(1+\mu _3)(1+\eta )\big )^2 \geqslant C^*(\mu _1^2+\mu _2^2+\mu _3^2), \end{aligned}$$

where

$$\begin{aligned} C^* = \frac{1}{3}\min \bigg \{\frac{c_{2\infty }+c_{3\infty }}{c_\infty }, \frac{c_{1\infty }^2}{c_{3\infty }^2(\mu _{\mathrm{max}}+2)^2}, \frac{c_{2\infty }^2}{c_{3\infty }^2(\mu _{\mathrm{max}}+2)^2}\mu _2^2\bigg \}. \end{aligned}$$

This proves (49) and completes the proof. $\quad \square$

## Convergence to Equilibrium for Complex-Balance Systems

One of the main assumptions of this paper is the detailed-balance condition (5). This condition was used extensively in the thermodynamic community and it leads to a natural entropy functional that is the core tool for the global existence analysis and the large-time asymptotics. However, the detailed-balance condition requires that the reaction system is reversible which is quite restrictive. In chemical reaction network theory, it is well known that there exists a much larger class of reaction systems, namely so-called *complex-balance systems* which also exhibits an entropy structure; see, e.g., [18, 23, 25] for reaction–diffusion systems. In this section, we show that the global existence and large-time behavior results can be extended to systems satisfying the complex-balance condition. We only highlight the differences of the proofs and present full proofs only when necessary:

Consider *n* constituents $A_i$ reacting in the following *N* reactions,

$$\begin{aligned} y_{1,a} A_1 + \cdots + y_{n,a} A_n \xrightarrow {k^a} y'_{1,a} A_1 + \cdots + y'_{n,a} A_n \quad \text{ for } a=1,\ldots ,N, \end{aligned}$$

where $k^a>0$ is the reaction rate constant and $y_{i,a}$, $y'_{i,a}\in \{0\}\cup [1,\infty )$ are the stoichiometric coefficients. We set ${\varvec{y}}_a=(y_{1,a},\ldots ,y_{n,a})$ and ${\varvec{y}}'_a=(y'_{1,a},\ldots , y'_{n,a})$. We denote by $\mathcal {C}=\{{\varvec{y}}_a,{\varvec{y}}'_a\}_{a=1,\ldots ,N}$ the set of all complexes. We use as in [18] the convention that the primed complexes ${\varvec{y}}_a'\in \mathcal {C}$ denote the product of the *a*th reaction, and the unprimed complexes ${\varvec{y}}_a\in \mathcal {C}$ denote the reactant. Note that it may happen that ${\varvec{y}}_a={\varvec{y}}'_b$ for some *a*, $b\in \{1,\ldots ,N\}$. This means that a complex can be a reactant for one reaction and a product for another reaction.

The Maxwell–Stefan diffusion system consists of equations (1), (3), and

54 $$\begin{aligned} r_i({\varvec{x}}) = M_i\sum _{a=1}^N k^a(y'_{i,a}-y_{i,a}){\varvec{x}}^{{\varvec{y}}_a}\quad \text{ with } {\varvec{x}}^{{\varvec{y}}_a} = \prod _{i=1}^n x_i^{y_{i,a}}. \end{aligned}$$

We assume again the conservation of total mass, expressed as

$$\begin{aligned} \sum _{i=1}^n r_i({\varvec{x}}) = 0. \end{aligned}$$

### Definition 1

(*Complex-balance condition*) A homogeneous equilibrium state ${\varvec{x}}_\infty$ is called a *complex-balance equilibrium* if for any ${\varvec{y}}\in \mathcal {C}$, it holds that

55 $$\begin{aligned} \sum _{a\in \{1,\ldots ,N\}:{\varvec{y}}_a={\varvec{y}}} k^a{\varvec{x}}_\infty ^{{\varvec{y}}_a} = \sum _{b\in \{1,\ldots ,N\}:{\varvec{y}}'_b={\varvec{y}}} k^b{\varvec{x}}_\infty ^{{\varvec{y}}_b}. \end{aligned}$$

Roughly speaking, ${\varvec{x}}_\infty$ is a complex-balance equilibrium if for any complex ${\varvec{y}}\in \mathcal {C}$ the total input into each complex balances the total flow out of the complex. The condition is weaker than detailed balance since it does not require each step in the forward reaction to be balanced by a reverse reaction. We say that system (1), (3), and (54) is a complex-balance system if it admits a positive complex-balance equilibrium. Already Boltzmann studied complex-balance systems in the context of kinetic theory, under the name of semi-detailed balance [4]. For chemical reaction systems, this condition was systematically studied in [22, 32].

The existence of global weak solutions to (1), (3), and (54) follows as in Section 2. We just have to verify that Lemma 7 also holds in the case of the reaction terms (54).

### Lemma 28

Let ${\varvec{x}}_\infty$ be a positive complex-balance equilibrium and let the entropy variable ${\varvec{w}}\in {\mathbb {R}}^{n-1}$ be defined by $w_i=\partial h/\partial \rho _i$, $i=1,\ldots ,n-1$, where *h* is given by (8). Then for all ${\varvec{x}}\in {\mathbb {R}}^n$, considered as a function of ${\varvec{w}}$,

$$\begin{aligned} \sum _{i=1}^{n-1}r_i({\varvec{x}})w_i \leqslant 0. \end{aligned}$$

### Proof

By (18) and definition (54) of $r_i$, we compute

$$\begin{aligned} \sum _{i=1}^{n-1}r_i({\varvec{x}})w_i&= \sum _{i=1}^n\frac{r_i({\varvec{x}})}{M_i}\ln \frac{x_i}{x_{i\infty }} = \sum _{i=1}^n\sum _{a=1}^N k^a(y_{i,a}'-y_{i,a}){\varvec{x}}^{{\varvec{y}}_a} \ln \frac{x_i}{x_{i\infty }} \\&= \sum _{a=1}^N k^a{\varvec{x}}^{{\varvec{y}}_a} \ln \frac{{\varvec{x}}^{{\varvec{y}}'_a-{\varvec{y}}}}{{\varvec{x}}_\infty ^{{\varvec{y}}'_a-{\varvec{y}}}} \\&= -\sum _{a=1}^N k^a{\varvec{x}}_\infty ^{{\varvec{y}}_a}\bigg \{ \frac{{\varvec{x}}^{{\varvec{y}}_a}}{{\varvec{x}}_\infty ^{{\varvec{y}}_a}} \ln \bigg (\frac{{\varvec{x}}^{{\varvec{y}}_a}}{{\varvec{x}}_\infty ^{{\varvec{y}}_a}}\biggl / \frac{{\varvec{x}}^{{\varvec{y}}'_a}}{{\varvec{x}}_\infty ^{{\varvec{y}}'_a}}\bigg ) - \frac{{\varvec{x}}^{{\varvec{y}}_a}}{{\varvec{x}}_\infty ^{{\varvec{y}}_a}} + \frac{{\varvec{x}}^{{\varvec{y}}'_a}}{{\varvec{x}}_\infty ^{{\varvec{y}}'_a}}\bigg \} \\&\phantom {xx}{}- \sum _{a=1}^N k^a{\varvec{x}}_\infty ^{{\varvec{y}}_a} \bigg (\frac{{\varvec{x}}^{{\varvec{y}}_a}}{{\varvec{x}}_\infty ^{{\varvec{y}}_a}} - \frac{{\varvec{x}}^{{\varvec{y}}'_a}}{{\varvec{x}}_\infty ^{{\varvec{y}}'_a}}\bigg ). \end{aligned}$$

The expression in the curly brackets $\{\cdots \}$ equals $\Psi ({\varvec{x}}^{{\varvec{y}}_a}/{\varvec{x}}_\infty ^{{\varvec{y}}_a},{\varvec{x}}^{{\varvec{y}}'_a}/ {\varvec{x}}_\infty ^{{\varvec{y}}'_a})$, where $\Psi (x,y)=x\ln (x/y)-x+y$ is a nonnegative function. Hence, the first expression on the right-hand side is nonpositive. We claim that the second expression vanishes. Then $\sum _{i=1}^{n-1}r_i({\varvec{x}})w_i\leqslant 0$. Indeed, by the complex-balance condition (55),

$$\begin{aligned} \sum _{a=1}^N k^a{\varvec{x}}_\infty ^{{\varvec{y}}_a} \bigg (\frac{{\varvec{x}}^{{\varvec{y}}_a}}{{\varvec{x}}_\infty ^{{\varvec{y}}_a}} - \frac{{\varvec{x}}^{{\varvec{y}}'_a}}{{\varvec{x}}_\infty ^{{\varvec{y}}'_a}}\bigg )&= \sum _{{\varvec{x}}\in \mathcal {C}}\bigg (\sum _{a:{\varvec{y}}_a={\varvec{y}}}k^a{\varvec{x}}^{{\varvec{y}}_a} - \sum _{b:{\varvec{y}}'_b={\varvec{y}}}k^b{\varvec{x}}_\infty ^{{\varvec{y}}_b} \frac{{\varvec{x}}^{{\varvec{y}}'_b}}{{\varvec{x}}_\infty ^{{\varvec{y}}'_b}}\bigg ) \\&= \sum _{{\varvec{y}}\in \mathcal {C}}\bigg ({\varvec{x}}^{{\varvec{y}}}\sum _{a:{\varvec{y}}_a={\varvec{y}}} k^a - \frac{{\varvec{x}}^{{\varvec{y}}}}{{\varvec{x}}_\infty ^{{\varvec{y}}}}\sum _{b:{\varvec{y}}'_b={\varvec{y}}} k^b{\varvec{x}}_\infty ^{{\varvec{y}}_b}\bigg ) \\&= \sum _{{\varvec{y}}\in \mathcal {C}}\frac{{\varvec{x}}^{{\varvec{y}}}}{{\varvec{x}}_\infty ^{{\varvec{y}}}} \bigg (\sum _{a:{\varvec{y}}_a={\varvec{y}}}k^a{\varvec{x}}_\infty ^{{\varvec{y}}_a} - \sum _{b:{\varvec{y}}_b'={\varvec{y}}}k^b{\varvec{x}}_\infty ^{{\varvec{y}}_b}\bigg ) = 0. \end{aligned}$$

This shows the claim and ends the proof. $\quad \square$

Next, we show the existence of a unique complex-balance equilibrium. For this, we denote as before by $\mathbb {W}=({\varvec{y}}'_a-{\varvec{y}}_a)_{a=1,\ldots ,N}\in {\mathbb {R}}^{n\times N}$ the Wegscheider matrix, set $m=\dim ({\text {ker}}\mathbb {W})>0$, and denote by ${\mathbb {Q}}\in {\mathbb {R}}^{m\times n}$ the matrix whose rows form a basis of ${\text {ker}}(\mathbb {W}^\top )$. As in Section 3.1, the conservation laws are given by

$$\begin{aligned} {\mathbb {Q}}\overline{{\varvec{c}}}(t) = {{\varvec{M}}^0}:= {\mathbb {Q}}\overline{{\varvec{c}}^0}, \quad t>0, \end{aligned}$$

and there exists ${\varvec{\zeta }}\in {\mathbb {R}}^{1\times m}$ such that ${\varvec{\zeta }}{\mathbb {Q}}={\varvec{M}}^\top$ and ${\varvec{\zeta }}{{\varvec{M}}^0}=1$.

### Proposition 29

(Existence of a complex-balance equilibrium) Let ${{\varvec{M}}^0}\in {\mathbb {R}}_+^m$ be an initial mass vector satisfying ${\varvec{\zeta }}{{\varvec{M}}^0}=1$. Then there exists a unique positive complex-balance equilibrium ${\varvec{x}}_\infty \in {\mathbb {R}}_+^n$ satisfying (55) and

56 $$\begin{aligned} {\mathbb {Q}}{\varvec{x}}_\infty = {{\varvec{M}}^0}\sum _{i=1}^n M_ix_{i\infty }, \quad \sum _{i=1}^n x_{i\infty }=1. \end{aligned}$$

The proof follows from the case of detailed balance with the help of the following lemma:

### Lemma 30

Let ${\varvec{x}}_\infty$ be a positive complex-balance equilibrium. Then the following two statements are equivalent:

(i) The vector ${\varvec{x}}_*\in {\mathbb {R}}_+^n$ is a complex-balance equilibrium.
(ii) It holds for all $a=1,\ldots ,N$ that
  $$\begin{aligned} \frac{{\varvec{x}}_*^{{\varvec{y}}_a}}{{\varvec{x}}_*^{{\varvec{y}}'_a}} = \frac{{\varvec{x}}_\infty ^{{\varvec{y}}_a}}{{\varvec{x}}_\infty ^{{\varvec{y}}'_a}}. \end{aligned}$$

### Proof

Let (ii) hold. We compute

$$\begin{aligned} \sum _{a:{\varvec{y}}_a={\varvec{y}}}k^a{\varvec{x}}_*^{{\varvec{y}}_a}&= \sum _{a:{\varvec{y}}_a={\varvec{y}}}k^a{\varvec{x}}_\infty ^{{\varvec{y}}_a} \frac{{\varvec{x}}_*^{{\varvec{y}}_a}}{{\varvec{x}}_\infty ^{{\varvec{y}}_a}} = \frac{{\varvec{x}}_*^{{\varvec{y}}}}{{\varvec{x}}_\infty ^{{\varvec{y}}}} \sum _{a:{\varvec{y}}_a={\varvec{y}}}k^a{\varvec{x}}_\infty ^{{\varvec{y}}_a} \\&= \frac{{\varvec{x}}_*^{{\varvec{y}}}}{{\varvec{x}}_\infty ^{{\varvec{y}}}} \sum _{b:{\varvec{y}}'_b={\varvec{y}}}k^b{\varvec{x}}_\infty ^{{\varvec{y}}_b} = \sum _{b:{\varvec{y}}'_b={\varvec{y}}}k^b{\varvec{x}}_\infty ^{{\varvec{y}}_b} \frac{{\varvec{x}}_*^{{\varvec{y}}'_b}}{{\varvec{x}}_\infty ^{{\varvec{y}}'_b}}. \end{aligned}$$

Taking into account (ii), it follows that

$$\begin{aligned} \sum _{a:{\varvec{y}}_a={\varvec{y}}}k^a{\varvec{x}}_*^{{\varvec{y}}_a} = \sum _{b:{\varvec{y}}'_b={\varvec{y}}}k^b{\varvec{x}}_\infty ^{{\varvec{y}}_b} \frac{{\varvec{x}}_*^{{\varvec{y}}_b}}{{\varvec{x}}_\infty ^{{\varvec{y}}_b}} = \sum _{b:{\varvec{y}}'_b={\varvec{y}}}k^b{\varvec{x}}_*^{{\varvec{y}}_b}, \end{aligned}$$

i.e., ${\varvec{x}}_*$ is a complex-balance equilibrium.

To show that (i) implies (ii), let ${\varvec{x}}_*$ be a complex-balance equilibrium. Then $r_i({\varvec{x}}_*)=0$ for all $i=1,\ldots ,n$, and the proof of Lemma 28 shows that

$$\begin{aligned} 0 = \sum _{i=1}^n\frac{r_i({\varvec{x}}_*)}{M_i}\ln \frac{x_{i*}}{x_{i\infty }} = -\sum _{a=1}^N k^a{\varvec{x}}_\infty ^{{\varvec{y}}_a}\Psi \bigg ( \frac{{\varvec{x}}_*^{{\varvec{y}}_a}}{{\varvec{x}}_\infty ^{{\varvec{y}}_a}}, \frac{{\varvec{x}}_*^{{\varvec{y}}'_a}}{{\varvec{x}}_\infty ^{{\varvec{y}}'_a}}\bigg ), \end{aligned}$$

where we recall that $\Psi (x,y)=x\ln (x/y)-x+y\geqslant 0$ and $\Psi (x,y)=0$ if and only if $x=y$. The last property implies that ${\varvec{x}}_*^{{\varvec{y}}_a}/{\varvec{x}}_\infty ^{{\varvec{y}}_a} = {\varvec{x}}_*^{{\varvec{y}}'_a}/{\varvec{x}}_\infty ^{{\varvec{y}}'_a}$, which is (ii). $\quad \square$

We prove a result similar to that one stated in Lemma 20.

### Lemma 31

The vector $\overline{{\varvec{\omega }}}=(\overline{c_1},\ldots ,\overline{c_n},\overline{c}) \in {\mathbb {R}}_+^{n+1}$ satisfies

57 $$\begin{aligned} \sqrt{\frac{\overline{{\varvec{\omega }}}}{{\varvec{\omega }}_\infty }}^{{\varvec{\mu }}^a} = \sqrt{\frac{\overline{{\varvec{\omega }}}}{{\varvec{\omega }}_\infty }}^{{\varvec{\nu }}^a} \quad \text{ for } \text{ all } a=1,\ldots ,N, \quad \widehat{{\mathbb {Q}}}\overline{{\varvec{\omega }}} = {\widehat{{\varvec{M}}}}^0, \end{aligned}$$

if and only if $\overline{{\varvec{\omega }}}={\varvec{\omega }}_\infty =(c_{1\infty },\ldots , c_{n\infty },c_\infty )$ and ${\varvec{x}}_\infty =(c_{1\infty }/c_\infty ,\ldots ,c_{n\infty }/c_\infty )$ is a complex-balance equilibrium. Here, $c_\infty =\sum _{i=1}^n c_{i\infty }$ and $\widehat{{\mathbb {Q}}}$ and ${\widehat{{\varvec{M}}}}^0$ are defined in (40).

### Proof

Set $x_i=\overline{c_i}/\overline{c}$ for $i=1,\ldots ,n$. Then the first equation in (57) implies that, using definition (22) of ${\varvec{\mu }}^a$ and ${\varvec{\nu }}^a$,

$$\begin{aligned} \prod _{i=1}^n \frac{\overline{c_i}^{y_{i,a}}}{c_{i\infty }^{y_{i,a}}} = \prod _{i=1}^n\frac{\overline{c_i}^{y'_{i,a}}}{c_{i\infty }^{y'_{i,a}}} \frac{\overline{c}^{\gamma ^a}}{c_\infty ^{\gamma ^a}}, \quad \text{ where } \gamma ^a = \sum _{i=1}^n(y_{i,a}-y'_{i,a}). \end{aligned}$$

This is equivalent to

$$\begin{aligned} \frac{{\varvec{x}}^{{\varvec{y}}_a}}{{\varvec{x}}_\infty ^{{\varvec{y}}_a}} = \frac{{\varvec{x}}^{{\varvec{y}}'_a}}{{\varvec{x}}_\infty ^{{\varvec{y}}'_a}}. \end{aligned}$$

We conclude from Lemma 30 that ${\varvec{x}}$ is a complex-balance equilibrium. Furthermore, we have

$$\begin{aligned} \sum _{i=1}^n M_ix_i = \frac{1}{\overline{c}}\sum _{i=1}^n M_i\overline{c_i} = \frac{1}{\overline{c}}. \end{aligned}$$

Thus, we deduce from the conservation law $\widehat{{\mathbb {Q}}}\overline{{\varvec{\omega }}} = {\widehat{{\varvec{M}}}}^0$ that

$$\begin{aligned} {\mathbb {Q}}{\varvec{x}} = \frac{1}{\overline{c}}{{\varvec{M}}^0}= {{\varvec{M}}^0}\sum _{i=1}^n M_ix_i. \end{aligned}$$

At this point, we can apply Proposition 29 to infer the existence of a unique vector ${\varvec{x}}={\varvec{x}}_\infty$ which implies that $\overline{{\varvec{\omega }}}={\varvec{\omega }}_\infty$. $\quad \square$

Finally, we show an inequality which is related to that one in Lemma 22.

### Lemma 32

There exists a nonconstructive constant $C>0$ such that

$$\begin{aligned} \sum _{a=1}^N\bigg (\sqrt{\frac{\overline{{\varvec{\omega }}}}{{\varvec{\omega }}_\infty }}^{{\varvec{\mu }}^a} - \sqrt{\frac{\overline{{\varvec{\omega }}}}{{\varvec{\omega }}_\infty }}^{{\varvec{\nu }}^a}\bigg )^2 \geqslant C\sum _{i=1}^{n+1}\big (\overline{\omega _i}^{1/2}-\omega _{i\infty }^{1/2}\big )^2 \end{aligned}$$

for all $\overline{{\varvec{\omega }}}\in {\mathbb {R}}_+^{n+1}$ satisfying $\widehat{{\mathbb {Q}}}\overline{{\varvec{\omega }}}={\widehat{{\varvec{M}}}}^0$.

### Proof

We proceed similarly as in the proofs of Lemmas 25 and 22. We need to show that

$$\begin{aligned} \lambda := \inf _{\overline{{\varvec{\omega }}}\in {\mathbb {R}}_+^{n+1}: \widehat{{\mathbb {Q}}}\overline{{\varvec{\omega }}}={\widehat{{\varvec{M}}}}^0} \frac{\sum _{a=1}^N\big (\sqrt{\overline{{\varvec{\omega }}}/{\varvec{\omega }}_\infty }^{{\varvec{\mu }}^a}- \sqrt{\overline{{\varvec{\omega }}}/{\varvec{\omega }}_\infty }^{{\varvec{\nu }}^a}\big )^2}{\sum _{i=1}^{n+1} \big (\overline{\omega _i}^{1/2}-\omega _{i\infty }^{1/2}\big )^2} > 0. \end{aligned}$$

In view of Lemma 31 and the absence of boundary equilibria, it holds $\lambda >0$ if and only if $\delta >0$, where

$$\begin{aligned} \delta&= \liminf _{\widehat{{\mathbb {Q}}}\overline{{\varvec{\omega }}}={\widehat{{\varvec{M}}}}^0,\,\overline{{\varvec{\omega }}} \rightarrow {\varvec{\omega }}_\infty } \frac{\sum _{a=1}^N\big (\sqrt{\overline{{\varvec{\omega }}}/{\varvec{\omega }}_\infty }^{{\varvec{\mu }}^a} - \sqrt{\overline{{\varvec{\omega }}}/{\varvec{\omega }}_\infty }^{{\varvec{\nu }}^a}\big )^2}{\sum _{i=1}^{n+1} \big (\overline{\omega _i}^{1/2}-\omega _{i\infty }^{1/2}\big )^2} \\&= \liminf _{\widehat{{\mathbb {Q}}}\overline{{\varvec{\omega }}}={\widehat{{\varvec{M}}}}^0,\,\overline{{\varvec{\omega }}} \rightarrow {\varvec{\omega }}_\infty } \frac{2\sum _{a=1}^N\big (\sum _{i=1}^{n+1}(y_{i,a}-y'_{i,a})(\overline{\omega _i} - \omega _{i\infty })\omega _{i\infty }^{-1}\big )^2}{\sum _{i=1}^{n+1}(\overline{\omega _i} -\omega _{i\infty })^2\omega _{i\infty }^{-1}}. \end{aligned}$$

This follows from a Taylor expansion as in the proof of Lemma 25. Now, we can follow exactly the arguments in the proof of Lemma 22 to infer that $\delta >0$ and consequently $\lambda >0$, finishing the proof. $\quad \square$

The results in this subsection are sufficient to apply the proof of Theorem 1, thus leading to the following main theorem.

### Theorem 33

(Convergence to equilibrium for complex-balance systems)

Let Assumptions (A1) and (A3) hold and let system (1), (54) be complex balanced. Fix an initial mass vector ${{\varvec{M}}^0}\in {\mathbb {R}}_+^m$ satisfying ${\varvec{\zeta }}{{\varvec{M}}^0}= 1$. Then

(i) There exists a global bounded weak solution ${\varvec{\rho }}=(\rho _1,\ldots ,\rho _n)^\top$ to (1), (3) with reaction terms (54) in the sense of Theorem 4;
(ii) There exists a unique positive complex-balance equilibrium ${\varvec{x}}_\infty \in {\mathbb {R}}_+^n$ satisfying (55) and (56);
(iii) Assume in addition that system (1), (54) has no boundary equilibria. Then there exist constants $C>0$ and $\lambda >0$, which are constructive up to a finite-dimensional inequality, such that if ${\varvec{\rho }}^0$ satisfies additionally $\mathbb Q\int _{\Omega }{\varvec{c}}^0\hbox {d}z = {{\varvec{M}}^0}$, the following exponential convergence to equilibrium holds:
  $$\begin{aligned} \sum _{i=1}^n\Vert x_i(t)-x_{i\infty }\Vert _{L^p(\Omega )} \leqslant Ce^{-\lambda t/(2p)} E[{\varvec{x}}^0|{\varvec{x}}_\infty ]^{1/(2p)} ,\quad t>0, \end{aligned}$$
  where $1\leqslant p <\infty$, $x_i=\rho _i/(cM_i)$ with $c=\sum _{i=1}^n\rho _i/M_i$, and $E[{\varvec{x}}|{\varvec{x}}_\infty ]$ is the relative entropy defined in (9), ${\varvec{\rho }}$ is the solution constructed in (i), and ${\varvec{x}}_\infty$ is constructed in (ii).

## Appendix A. Proof of Lemma 21

The proof of Lemma 21 is partially inspired by the proof of Lemma 2.7 in [25]. We divide the proof into two steps, which are presented in Lemmas 34 and 35. For convenience, we set $W_i:=\omega _i^{1/2}$ for $i=1,\ldots ,n+1$ and use the notation

$$\begin{aligned} {\varvec{W}} = (W_1,\ldots ,W_{n+1}), \quad \overline{{\varvec{W}}} = (\overline{W_1},\ldots ,\overline{W}_{n+1}). \end{aligned}$$

Moreover, we define

$$\begin{aligned} \delta _i(x) = W_i(x) - \overline{W_i} = W_i(x) - \int _\Omega W_i\hbox {d}z, \quad x\in \Omega ,\ i=1,\ldots ,n+1. \end{aligned}$$

### Lemma 34

There exists a constant $C>0$ depending on $\Omega$, *n*, *N*, $k_f^a$, and $k_b^a$ ($a=1,\ldots ,N$) such that

58 $$\begin{aligned} \widetilde{D}[{\varvec{\omega }}]\geqslant C\sum _{a=1}^N\Big ((k_f^a)^{1/2} \overline{{\varvec{W}}}^{{\varvec{\mu }}^a} - (k_b^a)^{1/2}\overline{{\varvec{W}}}^{{\varvec{\nu }}^a}\Big )^2, \end{aligned}$$

where $\widetilde{D}$ is defined in (39).

### Proof

We use the elementary inequality $(x-y)\ln (x/y)\geqslant 4(\sqrt{x}-\sqrt{y})^2$ to obtain

$$\begin{aligned} \int _\Omega \big (k_f^a{\varvec{\omega }}^{{\varvec{\mu }}^a}-k_b^a{\varvec{\omega }}^{{\varvec{\nu }}^a}\big ) \ln \frac{k_f^a{\varvec{\omega }}^{{\varvec{\mu }}^a}}{k_b^a{\varvec{\omega }}^{{\varvec{\nu }}^a}}\hbox {d}z \geqslant 4\int _\Omega \big ( (k_f^a)^{1/2}{\varvec{W}}^{{\varvec{\mu }}^a}-(k_b^a)^{1/2}{\varvec{W}}^{{\varvec{\nu }}^a}\big )^2 \hbox {d}z. \end{aligned}$$

This gives

$$\begin{aligned} \widetilde{D}[{\varvec{\omega }}] \geqslant \sum _{i=1}^{n+1}\Vert \nabla W_i\Vert _{L^2(\Omega )}^2 + 4\sum _{i=1}^{n+1}\big \Vert (k_f^a)^{1/2}{\varvec{W}}^{{\varvec{\mu }}^a}-(k_b^a)^{1/2}{\varvec{W}}^{{\varvec{\nu }}^a}\big \Vert _{L^2(\Omega }^2. \end{aligned}$$

The Poincaré inequality

$$\begin{aligned} \Vert \nabla W_i\Vert _{L^2(\Omega )}^2 \geqslant C_P\Vert \delta _i\Vert _{L^2(\Omega )}^2 \end{aligned}$$

then shows that

59 $$\begin{aligned} \widetilde{D}[{\varvec{\omega }}] \geqslant C_P\sum _{i=1}^{n+1}\Vert \delta _i\Vert _{L^2(\Omega )}^2 + 4\sum _{i=1}^{n+1}\big \Vert (k_f^a)^{1/2}{\varvec{W}}^{{\varvec{\mu }}^a}-(k_b^a)^{1/2}{\varvec{W}}^{{\varvec{\nu }}^a}\big \Vert _{L^2(\Omega )}^2. \end{aligned}$$

Let $L>0$. We split $\Omega$ into the two domains

$$\begin{aligned} \Omega _L = \big \{x\in \Omega :|\delta _i(x)|\leqslant L \text{ for } i=1,\ldots ,n+1\big \}, \quad \Omega _L^c = \Omega \backslash \Omega _L. \end{aligned}$$

By Taylor expansion, we may write $W_i^{\mu _i^a}=(\overline{W_i}+\delta _i)^{\mu _i^a} = \overline{W_i}^{\mu _i^a} + R_i^*(\overline{W_i},\delta _i)\delta _i$, where $R_i^*$ depends continuously on $\overline{W_i}$ and $\delta _i$. Therefore,

$$\begin{aligned} \big \Vert (k_f^a)^{1/2}&{\varvec{W}}^{{\varvec{\mu }}^a} - (k_b^a)^{1/2}{\varvec{W}}^{{\varvec{\nu }}^a}\big \Vert _{L^2(\Omega )}^2 \\&\geqslant \int _{\Omega _L}\bigg |(k_f^a)^{1/2}\prod _{i=1}^{n+1} (\overline{W_i}+\delta _i)^{\mu _i^a} - (k_b^a)^{1/2}\prod _{i=1}^{n+1}(\overline{W_i}+\delta _i)^{\nu _i^a}\bigg |^2 \hbox {d}z \\&= \int _{\Omega _L}\bigg |(k_f^a)^{1/2}\prod _{i=1}^{n+1}\big (\overline{W_i}^{\mu _i^a} + R_i^*\delta _i\big ) - (k_b^a)^{1/2}\prod _{i=1}^{n+1}\big (\overline{W_i}^{\nu _i^a} + R_i^*\delta _i\big )\bigg |^2 \hbox {d}z \\&= \int _{\Omega _L}\bigg |(k_f^a)^{1/2}\overline{{\varvec{W}}}^{{\varvec{\mu }}^a} - (k_b^a)^{1/2}\overline{{\varvec{W}}}^{{\varvec{\nu }}^a} + Q^*\sum _{i=1}^{n+1}\delta _i \bigg |^2 \hbox {d}z, \end{aligned}$$

where $Q^*$ depends continously on $R_1^*,\ldots ,R_{n+1}^*$ and $\delta _1,\ldots ,\delta _{n+1}$. With the inequalities $(x+y)^2\geqslant \frac{1}{2}(x^2-y^2)$ and $(\sum _{i=1}^{n+1}x_i)^2 \leqslant (n+1)\sum _{i=1}^{n+1}x_i^2$, we estimate

$$\begin{aligned} \big \Vert (k_f^a)^{1/2}&{\varvec{W}}^{{\varvec{\mu }}^a} - (k_b^a)^{1/2}{\varvec{W}}^{{\varvec{\nu }}^a}\big \Vert _{L^2(\Omega )}^2 \\&\geqslant \frac{1}{2}|\Omega _L|\Big ((k_f^a)^{1/2}\overline{{\varvec{W}}}^{{\varvec{\mu }}^a} - (k_b^a)^{1/2}\overline{{\varvec{W}}}^{{\varvec{\nu }}^a}\Big )^2 - \int _{\Omega _L}(Q^*)^2(n+1)\sum _{i=1}^{n+1}|\delta _i|^2 \hbox {d}z \\&\geqslant \frac{1}{2}|\Omega _L|\Big ((k_f^a)^{1/2}\overline{{\varvec{W}}}^{{\varvec{\mu }}^a} - (k_b^a)^{1/2}\overline{{\varvec{W}}}^{{\varvec{\nu }}^a}\Big )^2 - C(L)(n+1)\sum _{i=1}^{n+1}\Vert \delta _i\Vert _{L^2(\Omega )}^2, \end{aligned}$$

where we used the bounds $|\delta _i|\leqslant L$ in $\Omega _L$ and $\overline{W_i}\leqslant C$ in $\Omega$ to estimate $Q^*$. Summing over $a=1,\ldots ,N$, this gives

60 $$\begin{aligned} \sum _{a=1}^N\big \Vert (k_f^a)^{1/2}{\varvec{W}}^{{\varvec{\mu }}^a} - (k_b^a)^{1/2}{\varvec{W}}^{{\varvec{\nu }}^a}\big \Vert _{L^2(\Omega )}^2&\geqslant \frac{1}{2}|\Omega _L|\sum _{a=1}^N\Big ((k_f^a)^{1/2}\overline{{\varvec{W}}}^{{\varvec{\mu }}^a} - (k_b^a)^{1/2}\overline{{\varvec{W}}}^{{\varvec{\nu }}^a}\Big )^2 \nonumber \\&\phantom {xx}{}- C(L)N(n+1)\sum _{i=1}^{n+1}\Vert \delta _i\Vert _{L^2(\Omega )}^2. \end{aligned}$$

In $\Omega _L^c$, we wish to estimate $\Vert \delta _i\Vert _{L^2(\Omega )}$ from below. For this, we observe that

$$\begin{aligned} \sum _{a=1}^N\Big ((k_f^a)^{1/2}\overline{{\varvec{W}}}^{{\varvec{\mu }}^a} - (k_b^a)^{1/2}\overline{{\varvec{W}}}^{{\varvec{\nu }}^a}\Big )^2 \leqslant C. \end{aligned}$$

Then, since $\sum _{i=1}^{n+1}|\delta _i|\geqslant L$ on $\Omega _L^c$,

61 $$\begin{aligned} \sum _{i=1}^{n+1}\Vert \delta _i\Vert _{L^2(\Omega )}^2&\geqslant \sum _{i=1}^{n+1}\int _{\Omega _L^c}|\delta _i|^2 \hbox {d}z \geqslant \frac{1}{n+1}\int _{\Omega _L^c}\bigg (\sum _{i=1}^{n+1}|\delta _i|\bigg )^2 \hbox {d}z \nonumber \\&\geqslant \frac{L^2|\Omega _L^c|}{n+1} \geqslant \frac{L^2|\Omega _L^c|}{(n+1)C} \sum _{a=1}^N\Big ((k_f^a)^{1/2}\overline{{\varvec{W}}}^{{\varvec{\mu }}^a} - (k_b^a)^{1/2}\overline{{\varvec{W}}}^{{\varvec{\nu }}^a}\Big )^2. \end{aligned}$$

Inserting (60) and (61) into (59), it follows for any $\theta \in (0,1)$ that

$$\begin{aligned} \widetilde{D}[{\varvec{\omega }}]&\geqslant C_P\sum _{i=1}^{n+1}\Vert \delta _i\Vert _{L^2(\Omega )}^2 + 4\theta \sum _{i=1}^{n+1}\big \Vert (k_f^a)^{1/2}{\varvec{W}}^{{\varvec{\mu }}^a}-(k_b^a)^{1/2}{\varvec{W}}^{{\varvec{\nu }}^a} \big \Vert _{L^2(\Omega )}^2 \\&\geqslant \frac{C_P}{2}\sum _{i=1}^{n+1}\Vert \delta _i\Vert _{L^2(\Omega )}^2 + \frac{C_P}{2}\frac{L^2|\Omega _L^c|}{(n+1)C} \sum _{a=1}^N\Big ((k_f^a)^{1/2}\overline{{\varvec{W}}}^{{\varvec{\mu }}^a} - (k_b^a)^{1/2}\overline{{\varvec{W}}}^{{\varvec{\nu }}^a}\Big )^2 \\&\quad + 2\theta |\Omega _L|\sum _{a=1}^N\Big ((k_f^a)^{1/2}\overline{{\varvec{W}}}^{{\varvec{\mu }}^a} - (k_b^a)^{1/2}\overline{{\varvec{W}}}^{{\varvec{\nu }}^a}\Big )^2 - 4\theta C(L)(n+1)\sum _{i=1}^{n+1}\Vert \delta _i\Vert _{L^2(\Omega )}^2 \\&\geqslant C\sum _{a=1}^N\Big ((k_f^a)^{1/2}\overline{{\varvec{W}}}^{{\varvec{\mu }}^a} - (k_b^a)^{1/2}\overline{{\varvec{W}}}^{{\varvec{\nu }}^a}\Big )^2, \end{aligned}$$

where we have chosen $\theta >0$ sufficiently small in the last step. This finishes the proof. $\quad \square$

### Lemma 35

There exists a constant $C>0$ depending on $\Omega$, *n*, *N*, $k_f^a$, and $k_b^a$ ($a=1,\ldots ,N$) such that

62 $$\begin{aligned} \begin{aligned}&\sum _{i=1}^{n+1}|\nabla \omega _i^{1/2}|^2 \hbox {d}z + \sum _{a=1}^N\Big ((k_f^a)^{1/2}\overline{{\varvec{W}}}^{{\varvec{\mu }}^a} - (k_b^a)^{1/2}\overline{{\varvec{W}}}^{{\varvec{\nu }}^a}\Big )^2 \\&\quad \geqslant C\sum _{a=1}^N\Big ((k_f^a)^{1/2}\sqrt{\overline{{\varvec{\omega }}}}^{{\varvec{\mu }}^a} - (k_b^a)^{1/2}\sqrt{\overline{{\varvec{\omega }}}}^{{\varvec{\nu }}^a}\Big )^2. \end{aligned} \end{aligned}$$

### Proof

It follows from

$$\begin{aligned} \Vert \delta _i\Vert _{L^2(\Omega )}^2 = \Vert W_i-\overline{W_i}\Vert _{L^2(\Omega )}^2 = \overline{\omega _i} - \overline{W_i}^2 = \big (\sqrt{\overline{\omega _i}}-\overline{W_i}\big ) \big (\sqrt{\overline{\omega _i}}+\overline{W_i}\big ) \end{aligned}$$

that

$$\begin{aligned} \overline{W_i} = \sqrt{\overline{\omega _i}} - Z_i\Vert \delta _i\Vert _{L^2(\Omega )}, \quad \text{ where } Z_i=\frac{\Vert \delta _i\Vert _{L^2(\Omega )}}{\sqrt{\overline{\omega _i}} +\overline{W_i}}\geqslant 0. \end{aligned}$$

Since

$$\begin{aligned} Z_i^2 = \frac{\Vert \delta _i\Vert _{L^2(\Omega )}^2}{(\sqrt{\overline{\omega _i}} +\overline{W_i})^2} = \frac{\overline{\omega _i}-\overline{W_i}^2}{(\sqrt{\overline{\omega _i}} +\overline{W_i})^2} = \frac{\sqrt{\overline{\omega _i}}-\overline{W_i}}{\sqrt{\overline{\omega _i}} +\overline{W_i}} \leqslant 1, \end{aligned}$$

we infer that $0\leqslant Z_i\leqslant 1$.

We continue by performing a Taylor expansion:

$$\begin{aligned} \overline{{\varvec{W}}}^{{\varvec{\mu }}^a} = \prod _{i=1}^{n+1}\big (\sqrt{\overline{\omega _i}} - Z_i\Vert \delta _i\Vert _{L^2(\Omega )} \big )^{\mu _i^a} = \prod _{i=1}^{n+1}\big (\sqrt{\overline{\omega _i}}^{\mu _i^a} + R_i^*\Vert \delta _i\Vert _{L^2(\Omega )}\big ), \end{aligned}$$

where $R_i^*$ depends continuously on $Z_i$ and $\Vert \delta _i\Vert _{L^2(\Omega )}$. Therefore, with another function $S^*$ depending continuously on $Z_i$ and $\Vert \delta _i\Vert _{L^2(\Omega )}$,

$$\begin{aligned} \overline{{\varvec{W}}}^{{\varvec{\mu }}^a} = \sqrt{\overline{{\varvec{\omega }}}}^{{\varvec{\mu }}^a} + S^*\sum _{i=1}^{n+1}\Vert \delta _i\Vert _{L^2(\Omega )}. \end{aligned}$$

This shows that

$$\begin{aligned} \sum _{a=1}^N&\Big ((k_f^a)^{1/2}\overline{{\varvec{W}}}^{{\varvec{\mu }}^a} - (k_b^a)^{1/2}\overline{{\varvec{W}}}^{{\varvec{\nu }}^a}\Big )^2 \\&= \sum _{a=1}^N\bigg ((k_f^a)^{1/2}\sqrt{\overline{{\varvec{\omega }}}}^{{\varvec{\mu }}^a} - (k_b^a)^{1/2}\sqrt{\overline{{\varvec{\omega }}}}^{{\varvec{\nu }}^a} + \big ((k_f^a)^{1/2}-(k_b^a)^{1/2}\big ) S^*\sum _{i=1}^{n+1}\Vert \delta _i\Vert _{L^2(\Omega )}\bigg )^2 \\&\geqslant \frac{1}{2}\sum _{a=1}^N\Big ((k_f^a)^{1/2}\sqrt{\overline{{\varvec{\omega }}}}^{{\varvec{\mu }}^a} - (k_b^a)^{1/2}\sqrt{\overline{{\varvec{\omega }}}}^{{\varvec{\nu }}^a}\Big )^2 - C(n,N)(S^*)^2\sum _{i=1}^{n+1}\Vert \delta _i\Vert _{L^2(\Omega )}^2. \end{aligned}$$

Then, by the Poincaré inequality with constant $C_P$, for some $\theta \in (0,1)$,

$$\begin{aligned} \sum _{i=1}^{n+1}|\nabla \omega _i^{1/2}|^2 \hbox {d}z&+ \sum _{a=1}^N\Big ((k_f^a)^{1/2}\overline{{\varvec{W}}}^{{\varvec{\mu }}^a} - (k_b^a)^{1/2}\overline{{\varvec{W}}}^{{\varvec{\nu }}^a}\Big )^2 \\&\geqslant C_P\sum _{i=1}^{n+1}\Vert \delta _i\Vert _{L^2(\Omega )}^2 + \theta \sum _{a=1}^N\Big ((k_f^a)^{1/2}\sqrt{\overline{{\varvec{\omega }}}}^{{\varvec{\mu }}^a} - (k_b^a)^{1/2}\sqrt{\overline{{\varvec{\omega }}}}^{{\varvec{\nu }}^a}\Big )^2 \\&\geqslant C_P\sum _{i=1}^{n+1}\Vert \delta _i\Vert _{L^2(\Omega )}^2 + \frac{\theta }{2} \sum _{a=1}^N\Big ((k_f^a)^{1/2}\sqrt{\overline{{\varvec{\omega }}}}^{{\varvec{\mu }}^a} - (k_b^a)^{1/2}\sqrt{\overline{{\varvec{\omega }}}}^{{\varvec{\nu }}^a}\Big )^2 \\&\quad - \theta C(n,N)(S^*)^2\sum _{i=1}^{n+1}\Vert \delta _i\Vert _{L^2(\Omega )}^2 \\&\geqslant \frac{\theta }{2} \sum _{a=1}^N\Big ((k_f^a)^{1/2}\sqrt{\overline{{\varvec{\omega }}}}^{{\varvec{\mu }}^a} - (k_b^a)^{1/2}\sqrt{\overline{{\varvec{\omega }}}}^{{\varvec{\nu }}^a}\Big )^2. \end{aligned}$$

The last step follows after choosing $\theta >0$ sufficiently small. This is possible since $S^*$ is bounded. The proof is complete. $\quad \square$

### Proof of Lemma 21

Applying first (58) and then (62) leads to

$$\begin{aligned} \widetilde{D}[{\varvec{\omega }}]&\geqslant \frac{C}{2}\sum _{i=1}^{n+1}\int _\Omega |\nabla \omega _i^{1/2}|^2 \hbox {d}z \\&\quad + \frac{C}{2}\bigg (\sum _{i=1}^{n+1}\int _\Omega |\nabla \omega _i^{1/2}|^2 \hbox {d}z + \sum _{a=1}^N\int _\Omega \big (k_f^a{\varvec{\omega }}^{{\varvec{\mu }}^a} -k_b^a{\varvec{\omega }}^{{\varvec{\nu }}^a}\big ) \ln \frac{k_f^a{\varvec{\omega }}^{{\varvec{\mu }}^a}}{k_b^a{\varvec{\omega }}^{{\varvec{\nu }}^a}}\hbox {d}z\bigg ) \\&\geqslant \frac{C}{2}\sum _{i=1}^{n+1}\int _\Omega |\nabla \omega _i^{1/2}|^2 \hbox {d}z + C\sum _{a=1}^N\Big ((k_f^a)^{1/2}\overline{{\varvec{W}}}^{{\varvec{\mu }}^a} - (k_b^a)^{1/2}\overline{{\varvec{W}}}^{{\varvec{\nu }}^a}\Big )^2 \\&\geqslant C\sum _{a=1}^N\Big ((k_f^a)^{1/2}\sqrt{\overline{{\varvec{\omega }}}}^{{\varvec{\mu }}^a} - (k_b^a)^{1/2}\sqrt{\overline{{\varvec{\omega }}}}^{{\varvec{\nu }}^a}\Big )^2. \end{aligned}$$

The proof is finished.
